# *Treponema pallidum* suppressive microenvironments and central nervous system damage: from mechanisms to tools and prevention

**DOI:** 10.3389/fimmu.2026.1866420

**Published:** 2026-09-15

**Authors:** Muze Huang, Shiyuan Wu, Zi-Han Shen, Sarena Jiayao Zhang, Yaw-syan Fu, Jingyi Wu

**Affiliations:** 1 School of Medicine, Xiamen University, Xiamen, Fujian, China; 2 Center of Clinical Laboratory, Zhongshan Hospital Xiamen University, School of Medicine Xiamen, Xiamen, Fujian, China; 3 Xiamen University, Xiamen, China; and Institute of Infectious Disease, School of Medicine, Xiamen University, Xiamen, Fujian, China; 4 School of Basic Medicine, Fujian Medical University, Fuzhou, Fujian, China; 5 School of Stomatology, Xiamen Medical College, Xiamen, Fujian, China; 6 School of Clinical Medicine, Xiamen Medical College, Xiamen, Fujian, China; 7 University of Tsukuba, Tsukuba, Ibaraki, Japan; 8 Department of Anatomy, School of Basic Medical Science, Xiamen Medical College, Xiamen, Fujian, China

**Keywords:** blood-brain barrier, immune evasion, neurosyphilis, suppressive microenvironments, *Treponema pallidum*

## Abstract

The global resurgence of syphilis, marked by a dramatic 150% increase in cases over the last decade and the rise of the dominant SS14-Ω strain cluster, represents a critical threat to public health. This review explores the complex molecular mechanisms by which *Treponema pallidum* functions as a “stealth pathogen,” establishing suppressive chemotactic microenvironments that facilitate neuroinvasion and persistent central nervous system (CNS) damage. We delineate a biomechanical and molecular cascade wherein the spirochete utilizes its unique “naked” outer membrane and hypervariable *Treponema pallidum* repeat protein K (TprK) to bypass host immune surveillance. Furthermore, we detail how the pathogen is increasingly understood to breach the blood-brain barrier (BBB) through the induction of pro-inflammatory signals like interleukin-1β (IL-1β) and the recently identified upregulation of ADAMTS5, which degrades the endothelial glycocalyx. Addressing the significance of recent advances, this review highlights the transition from traditional animal models to sophisticated *in vitro* genetic engineering, which has begun to overcome historical research bottlenecks. We conclude that while standard penicillin therapy effectively achieves serological cure, it is often insufficient to arrest or reverse the established neuro-inflammatory cascades that lead to permanent cognitive and neuropsychiatric impairments. Future perspectives emphasize the necessity of proactive prophylactic models like doxycycline post-exposure prophylaxis (DoxyPEP) and the identification of novel drug targets, such as the extracellular loops of outer membrane proteins, to disrupt immune evasion and may help mitigate irreversible neurological sequelae.

## 1 Introduction

*Treponema pallidum* is one of the oldest known pathogens to affect humans, and recent paleopathological evidence suggests it has existed for over 5,500 years. Even today, it remains a serious threat to public health because it is so good at hiding from the immune system and staying dormant for long periods (1–3). Although we have had effective antibiotics since the middle of the 20th century, this spirochete has made a sudden and dramatic comeback across the globe in recent decades (4). Recent data shows a very heavy burden, with an estimated 12 million new infections every year and at least 36 million people currently infected worldwide (5, 6). This trend is particularly pronounced to see in high-income countries, where the number of syphilis cases has jumped by more than 150% in the last ten years (7). This resurgence is even more difficult to manage because of a dominant pandemic strain cluster called SS14-Ω. This group grew very quickly following a population “bottleneck” that occurred in the late 1990s (4, 7). Often called a “stealth pathogen,” *Treponema pallidum* uses its unique outer membrane structure to help it spread early and dodge the host’s immune signals (8, 9). If a person is not treated, the bacteria can invade the central nervous system (CNS) and lead to severe brain tissue loss (1, 10). On top of this, the rising number of strains that can resist macrolide antibiotics underscores the inadequacy of existing control measures. We need to focus more on how the pathogen interacts with the host’s suppressive microenvironments to find better solutions (5, 11).

Compounding this public health crisis is the critical syndemic relationship between *Treponema pallidum* and other sexually transmitted pathogens, most notably the Human Immunodeficiency Virus (HIV). Epidemiological data consistently reveal a high prevalence of HIV co-infection among syphilis patients, driven by a potent bidirectional biological synergy (12, 13). The primary syphilitic chancre breaks epithelial barriers and recruits a dense influx of CD4+ T-cells and macrophages to the lesion site, thereby potentially enhancing the acquisition and transmission efficiency of HIV (14). Conversely, HIV-induced cellular immunodeficiency impairs the host’s ability to clear spirochetes, frequently leading to accelerated progression into neurosyphilis, higher titles of serological markers, and a propensity for atypical, aggressive clinical manifestations (15, 16). Understanding these co-infection dynamics is paramount, as they profoundly reshape the host’s systemic immune status and compromise standard therapeutic efficacies.

The primary challenge in understanding how *Treponema pallidum* causes disease is its reputation as an “elusive” pathogen. Several unique biological hurdles have historically blocked molecular research on this organism. A major issue is its incredibly small genome, measuring about 1.1 Mb. Unlike other invasive bacteria, it lacks the genes needed for many basic metabolic functions and common virulence factors, such as lipopolysaccharides (LPSs) or specialized secretion systems (17–19). This minimal genome makes the spirochete a “stealth pathogen.” Because it has very few proteins on its outer membrane, it can stay almost invisible to the host’s immune system for a long time (10, 17). Additionally, *Treponema pallidum* is extremely sensitive to its surroundings. For more than a hundred years, scientists could not grow it reliably in a lab setting, forcing them to use expensive and difficult animal models to keep strains alive (17, 18, 20). This long-standing struggle to achieve steady growth in the laboratory has made it very hard to use modern genetic engineering or transformation methods (21). While recent progress has finally allowed the bacteria to multiply over the long term using specialized systems with rabbit epithelial cells, the organism is still very difficult to work with. These complexities continue to slow down functional studies and the search for a vaccine (18, 20). As a result, figuring out how this sensitive spirochete moves through suppressive environments and triggers cell-killing signals remains a major goal in medical microbiology (10, 22).

Historically, before the dawn of the antibiotic era, the therapeutic landscape for syphilis was characterized by high systemic toxicity and severely limited efficacy. For centuries, mercury-based regimens were widely utilized both topically and orally; however, they frequently induced fatal heavy metal poisoning while offering negligible clearance of Treponema pallidum (23). In 1910, Paul Ehrlich revolutionized antimicrobial chemotherapy with the synthesis of Salvarsan (arsphenamine), hailed as the first “magic bullet” (24, 25). Although Salvarsan and its later derivative, Neosalvarsan, significantly improved clinical outcomes, their utilization was profoundly constrained by complex, long-term administration schedules, considerable hepatorenal toxicity, and incomplete cure rates in advanced stages (24, 26). To combat the devastating neurological manifestations of late-stage infection, Julius Wagner-Jauregg introduced “malaria therapy” in 1917, intentionally infecting neurosyphilis patients with malaria to induce sustained high fevers (>40 °C) capable of killing the heat-sensitive spirochetes (27). While this innovative approach earned the Nobel Prize in 1927, its inherent high mortality rate and immense systemic toll presented formidable clinical limitations. The subsequent advent and mass production of penicillin in the 1940s fundamentally rendered these hazardous modalities obsolete, establishing a highly effective and safe gold standard that reshaped syphilis management (28, 29).

This review explores the complex interaction between the suppressive environments created by *Treponema pallidum* and the resulting breakdown of the host’s cytotoxic signaling. By pulling together recent molecular evidence, we propose a two-part model to explain how this works. In this model, the immune subversion caused by the spirochete does more than just help the bacteria hide outside of cells for long periods; it also triggers the brain tissue loss seen in late-stage syphilis. Specifically, we look at how changes in cytokine levels and problems with white blood cell movement create “immune-evasive niches” inside the CNS. In this infectious neuroinflammatory context, this review proposes a model where these niches represent restricted anatomical or immunological microenvironments where *Treponema pallidum* exploits its low-antigenicity surface and alters local chemokine gradients, thereby impeding the recruitment and activation of functional effector immune cells. Ultimately, this summary offers new ideas on how a quiet, latent infection turns into a disease that destroys nerves. Our goal is to identify specific targets for treatment that could stop permanent neurological damage before it starts.

## 2 Stealth surface structures and immune evasion mechanisms

### 2.1 The barren outer membrane and passive immune evasion

The remarkable stealth pathogenicity of *Treponema pallidum* is fundamentally rooted in its unique ultrastructure. Specifically, its outer membrane acts like an immunological “black box” (30). Unlike typical Gram-negative bacteria, the *Treponema pallidum* outer membrane is known for its “paucity” of integral membrane proteins. It contains about 100 times less membrane-spanning protein than enteric bacteria like Escherichia coli (**Figure 1**) (31, 32). This minimalist molecular architecture creates a nearly naked lipid bilayer. Crucially, it lacks LPS, which is the main pathogen-associated molecular pattern (PAMP) that hosts use to identify Gram-negative bacteria (**Figure 1**) (32, 33). Because it lacks LPS, the spirochete successfully bypasses detection by host Toll-like receptors (TLRs). This absence of detection hinders early innate immune activation and stops the host from starting a strong inflammatory response (32, 33). This structural scarcity of surface antigens ensures the pathogen remains antigenically inert during the early stages of infection. This helps the bacteria spread through the system and establish a persistent infection before the host can put up an effective defense (30, 33).

**Figure 1:**
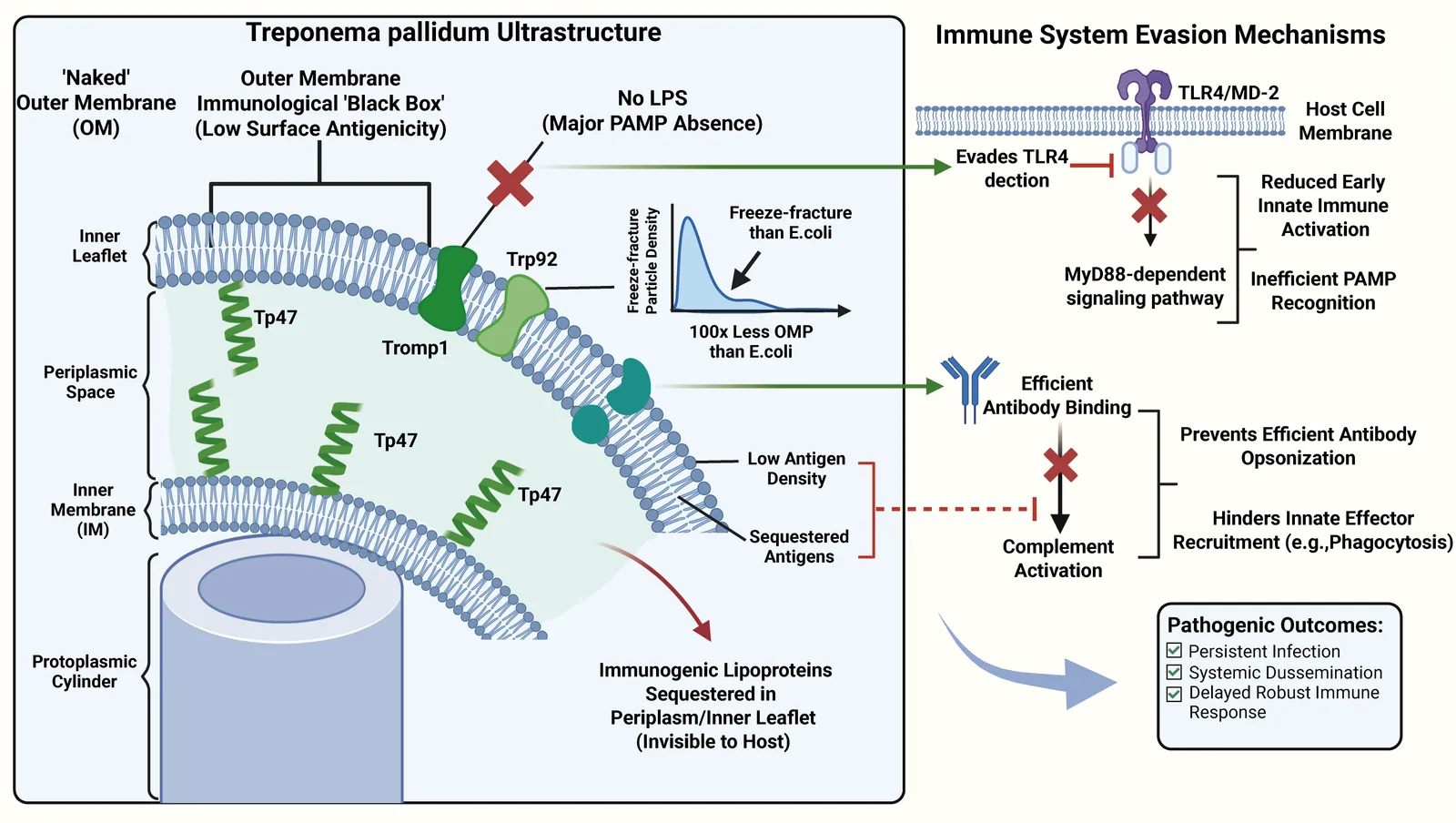
Molecular determinants of immune evasion and microenvironmental suppression. Treponema pallidum maintains persistence through a synergistic “stealth” strategy that integrates structural minimalism and stochastic genetic recombination. The ultrastructure (left) is characterized as an immunological “black box,” featuring an outer membrane with a 100-fold lower density of integral proteins compared to enteric bacteria and a complete absence of LPS, thereby evading TLR4-mediated innate detection. Simultaneously, the TprK system (right) drives chronic infection via non-reciprocal segmental gene conversion, stochastically pulling from 53 silent donor cassettes to alter seven surface-exposed variable (V) regions. These structural shifts abrogate antibody binding and opsonophagocytosis, while the expression of proteins like Tp92 further modulates the local niche by delaying neutrophil apoptosis, collectively facilitating a transition into asymptomatic latency.

Detailed freeze-fracture and deep-etching analyses confirm this unusual architecture. These methods reveal only a few uniformly sized intramembranous particles (IMPs) on the fracture faces of the outer membrane (34). These findings show that the major immunogenic lipoproteins, such as the 47-kDa immunogen (Tp47), are not actually surface-exposed. Instead, they are sequestered within the periplasmic space or anchored to the inner leaflet of the outer membrane. This structural sequestration shields them from host humoral defenses (**Figure 1**) (34, 35). Although researchers have found rare outer membrane proteins (TROMPs) like Tromp1 (TP0326), TprC/D, and Tp92 as surface-exposed orthologs of Gram-negative OMPs, their density is incredibly low. This scarcity ensures that the spirochete surface stays largely hidden from the host’s immune surveillance (31, 36, 37). On top of that, the pathogen uses phase variation to switch up its surface antigenic profile. For example, the stochastic variation of tprL expression is controlled by homopolymeric G sequences in its promoter, which helps it dodge the immune system (38). This strategic suppression of surface antigenicity stops antibodies from binding effectively. It also may impair the recruitment of innate effectors, such as macrophages for opsonophagocytosis. By avoiding these immune responses, *Treponema pallidum* can persist within the host milieu for a very long time (**Figure 1**) (30, 39). Notably, such strategies of physical masking or reducing the density of surface antigens to evade host surveillance are a common evolutionary theme observed across diverse microbial pathogens, including certain pathogenic fungi (40, 41). This mechanism of immune invisibility mirrors the structural masking strategies identified in fungal pathogens like Candida albicans.

### 2.2 Suppressive chemotaxis

*Treponema pallidum* enters latent and asymptomatic phases by skillfully changing the local microenvironment to quiet down the host’s inflammatory signals. A big part of this long-term infection is the pathogen’s ability to move into immune-privileged sites. This move lets the bacteria hide away from the host’s strong immune surveillance (42). This hiding is physically possible because of the spirochete’s unique ultrastructure. It has a “naked” outer membrane with very few surface proteins, acting like an immunological “black box” that stays under the radar (42). A major factor in this immune evasion is the *Treponema pallidum* repeat protein K (TprK) antigenic variation system. Through non-reciprocal segmental gene conversion at the TprK expression site, the bacteria constantly create new variants that dodge existing antibody pressures (43). This mechanism lets donor cassettes recombine into seven discrete variable regions. As a result, even when the host builds a humoral response, the treponemal population stays a moving target (44). Interestingly, research shows that this fast antigenic shift happens even without immune pressure. This provides a steady supply of new variants that keep the treponemal burden going without causing obvious clinical lesions (44). By mixing this structural “invisibility” with dynamic genetic shifting, the pathogen makes sure that early innate immune activation stays inefficient. This leads to a delayed and much weaker overall immune response (**Figure 1**) (42).

Furthermore, *Treponema pallidum* uses specific proteins like Tp92 to delay the apoptosis of human neutrophils. It does this by activating the extracellular signal-regulated kinase (ERK), phosphoinositide 3-kinase (PI3K)/protein kinase B (Akt), and nuclear factor kappa B (NF-κB) pathways, which changes the early inflammatory landscape to help the pathogen survive (37). By stopping these immune cells from dying when they should, *Treponema pallidum* may limit the release of more danger signals. This effectively mutes the host’s overall inflammatory alarm. While some interactions, like those with *Treponema pallidum* enolase, can trigger the secretion of pro-inflammatory cytokines such as IL-8 and IL-6 to help the bacteria spread (45), others promote M1 macrophage polarization. This drives vascular injury through the Janus kinase 1 (JAK1)-signal transducer and activator of transcription 1 (STAT1) signaling pathway (46). Together, these many-sided strategies let the spirochete exploit host responses while avoiding being cleared out (42). *Treponema pallidum* also interacts with endothelial cells and smooth muscle cells, triggering the production of monocyte chemoattractant protein-1 (MCP-1) and intercellular adhesion molecule-1 (ICAM-1). These molecules make it easier for the bacteria to migrate and invade across vascular barriers (47, 48). By mixing these methods—antigenic shift, structural sequestration, and interference with cellular signaling—*Treponema pallidum* creates a suppressive chemotactic niche (47). This niche keeps the host from recruiting effectors like phagocytes. This process allows for long-term survival and explains why latent infections in untreated hosts usually show no symptoms (42).

## 3 Antigenic variation driven by TprK

The chronic nature of *Treponema pallidum* infection is mainly is closely associated with the hypervariable outer membrane protein TprK. This protein helps the bacteria escape adaptive immune responses through continuous antigenic variation (44). This advanced survival process uses a non-reciprocal segmental gene conversion mechanism. In this process, genetic information from 53 silent chromosomal donor cassettes is randomly recombined into a single TprK expression site (43). This recombination creates massive sequence diversity within seven discrete variable (V) regions—V1 through V7—which are strategically positioned on the surface of the pathogen in a 3-base pair (bp) changing pattern (43, 49). Recent long-read sequencing analysis has further revealed that these V regions form complex full-length combined patterns that are remarkably heterogeneous, yet maintain stable gene length and GC content (50). Structural and modeling data show that TprK acts as an integral outer membrane porin, with these V regions placed to interact directly with the host immune system (43). These changes in structure effectively stop the binding of antibodies produced during infection, as antibodies elicited against one TprK variant show little to no cross-reactivity with others (51). These antibodies tend to target the variable regions rather than the β-barrel scaffolding of the protein (43). By dodging these specific antibodies, variant treponemes avoid opsonophagocytosis. This is a process where antibodies normally tag outer membrane antigens to help macrophages clear the bacteria (52). As a result, individual TprK variants escape the first immune response and start new disseminated lesions throughout the body. This mechanism is key to the progression from primary to secondary syphilis, as evidenced by the significant accumulation of TprK variants in patients transitioning between these stages (49, 50, 52). Research using a knockout strain (SS14-DC KO) with a broken variation system showed that without the ability to create new TprK sequences, the pathogen becomes much weaker and cannot overcome immune pressure (43). While immune selection clearly speeds up the buildup of these variants in immunocompetent hosts, gene conversion still happens at a steady baseline rate even without immune pressure. This has been proven through *in vitro* cultivation and studies in immunosuppressed rabbits (44, 53). Ultimately, the massive antigenic variability of TprK, which shows almost no sequence overlap between different clinical samples, creates a level of diversity that rivals the human adaptive immune system (54). This allows *Treponema pallidum* to compete directly with host defenses and establish persistent, lifelong infections (54).

## 4 Mechanisms of BBB traversal

### 4.1 Role of adhesins and endothelial stress

The pathogenesis of syphilis begins when *Treponema pallidum* infiltrates the host through microscopic abrasions in the squamous epithelium or urogenital mucous membranes, usually occurring during sexual intercourse (55, 56). Once it enters, this microaerophilic spirochete shows a strong tropism for local host tissues. It binds to the extracellular matrix (ECM) and starts binary fission. This local growth often leads to a primary indurated ulcer, or chancre. Under clinical observation, this lesion shows a dense infiltration of lymphocytes and plasma cells (57). A major virulence feature of *Treponema pallidum* is its ability to bypass local innate immune surveillance. It establishes a systemic infection almost immediately by spreading through the lymphatic and circulatory systems (55, 58). This rapid hematogenous spread depends heavily on the organism’s unique structural morphology. Its periplasmic flagella, located in the periplasmic space between the inner and outer membranes, create a powerful, corkscrew-like motility. This biomechanical trait allows the spirochete to move through highly viscous environments. It can cross complex tissue barriers that often block other bacterial pathogens (59). Although physical motility is essential, specialized surface-exposed proteases also significantly boost the invasion process. For example, the metalloprotease Tp0751 (pallilysin) serves two purposes. First, it helps with initial vascular adhesion by interacting with the 67-kDa laminin receptor (LamR) on host cells. Then, it undergoes host-mediated activation to degrade ECM components like laminin and fibrinogen (60). This specific proteolysis helps the spirochete break through the basement membrane. This step ensures it can move from the interstitial space into the vascular compartment (60).

Once inside the host’s vasculature, *Treponema pallidum* spreads using a specialized mechanism. It primarily crosses the endothelium through intercellular junctions. This paracellular pathway is a defining feature of treponemal invasion. It allows the spirochete to migrate across endothelial monolayers without causing the permanent structural damage or cell death usually seen with more cytolytic pathogens (47, 59). This transendothelial migration is an active process. It is supported by the pathogen-induced activation of host vascular endothelial cells. Specifically, *Treponema pallidum* surface lipoproteins trigger a pro-inflammatory response. This response leads to the up-regulation of ICAM-1 and boosts procoagulant activity. These changes effectively turn the endothelial surface into a more adhesive microenvironment that helps the bacteria dock (61, 62). Pathogenic molecules like TP0768 further may facilitate this molecular “remodeling” of the endothelium. Research shows that TP0768 promotes the adhesion and migration of monocytic cells to the vascular endothelium. It does this by triggering endoplasmic reticulum (ER) stress and activating the NF-κB/hypoxia-inducible factor 1-alpha (HIF-1α) signaling pathways (**Table 1**). This molecular remodeling and activation of signaling pathways collectively represent a state of endothelial stress, which facilitates the subsequent breach of the vascular barrier. Recent time-course transcriptomic analyses of brain microvascular endothelial cells exposed to the pathogen show that these interactions cause major changes in host pathways. These changes affect the ECM, integrin signaling, and Rho guanosine triphosphatase (GTPase) activity (63). Furthermore, the induction of the transcription factor Snail and the observed F-actin cellular contraction suggest that the spirochete may trigger an endothelial-to-mesenchymal transition (EndoMT). This phenotypic shift likely promotes vascular dysfunction. It makes it even easier for the spirochete to move through weakened intercellular junctions (63).

**Table 1:** Key pathogenic molecules of *Treponema pallidum* and their molecular mechanisms.

Pathogenic molecule	Molecular function & immunological outcome	Involved signaling pathways / receptors	Key references
TprK	Mediates high-level antigenic variation to evade antibody binding and opsonophagocytosis	7 variable (V) regions; 53 donor cassettes	(10)
Tp0751	Facilitates vascular adhesion and penetration of the blood-brain barrier	67-kDa laminin receptor	(60)
Tp92	Delays host neutrophil apoptosis to favor early pathogen survival and persistence	ERK, PI3K/Akt, and NF-κB pathways	(37, 70)
TP0768	Promotes monocytic cell adhesion and migration to vascular endothelial cells	ER stress; NF-κB/HIF-1α pathway	(71, 72)
Tp Enolase	Induces macrophage apoptosis and promotes inflammatory cytokine secretion (IL-6, IL-8)	ROS/NF-κB signaling axis	(45)
TP0954	Acts as a critical adhesin for binding to diverse host cells and distal neural pathways	Glioma cell lines and placental tissues	(73, 74)

This table integrates the various proteins mentioned in your review, linking specific mechanisms to their respective literature support.

Next, *Treponema pallidum* strategically exploits host hematological components, especially platelets, to boost its survival and extravasation potential during systemic dissemination. To survive early on in the hostile circulatory environment, the spirochete uses Tp92 (a surface-localized protein) to tune the host immune response. Specifically, it delays the apoptosis of neutrophils by activating the ERK, PI3K/Akt, and NF-κB signaling pathways (**Table 1**). This survival trick ensures that enough bacteria stay alive to seed distant sites. At the same time, *Treponema pallidum* can activate and pick out activated host platelets to interact with, a process that is notably reversible (**Figure 2**) (64). Detailed kinematic and darkfield microscopy analyses show that platelet-interacting treponemes move very differently compared to free-swimming ones. These findings suggest that *Treponema pallidum* might “hitchhike” on platelets. This helps it navigate the high-shear environment of the bloodstream and settle at distant vascular sites (64). These interactions do more than just help with physical transport. They also trigger a localized inflammatory response. This response involves secreting pro-inflammatory cytokines like IL-6, RANTES, and GM-CSF, which further weaken the defensive function of the endothelial barrier (63). By hijacking the natural role of platelets in controlling vascular permeability and immune signaling, *Treponema pallidum* effectively ensures it spreads quickly and widely throughout the system. This process sets the stage for the chronic complications of late-stage syphilis (58, 64).

**Figure 2:**
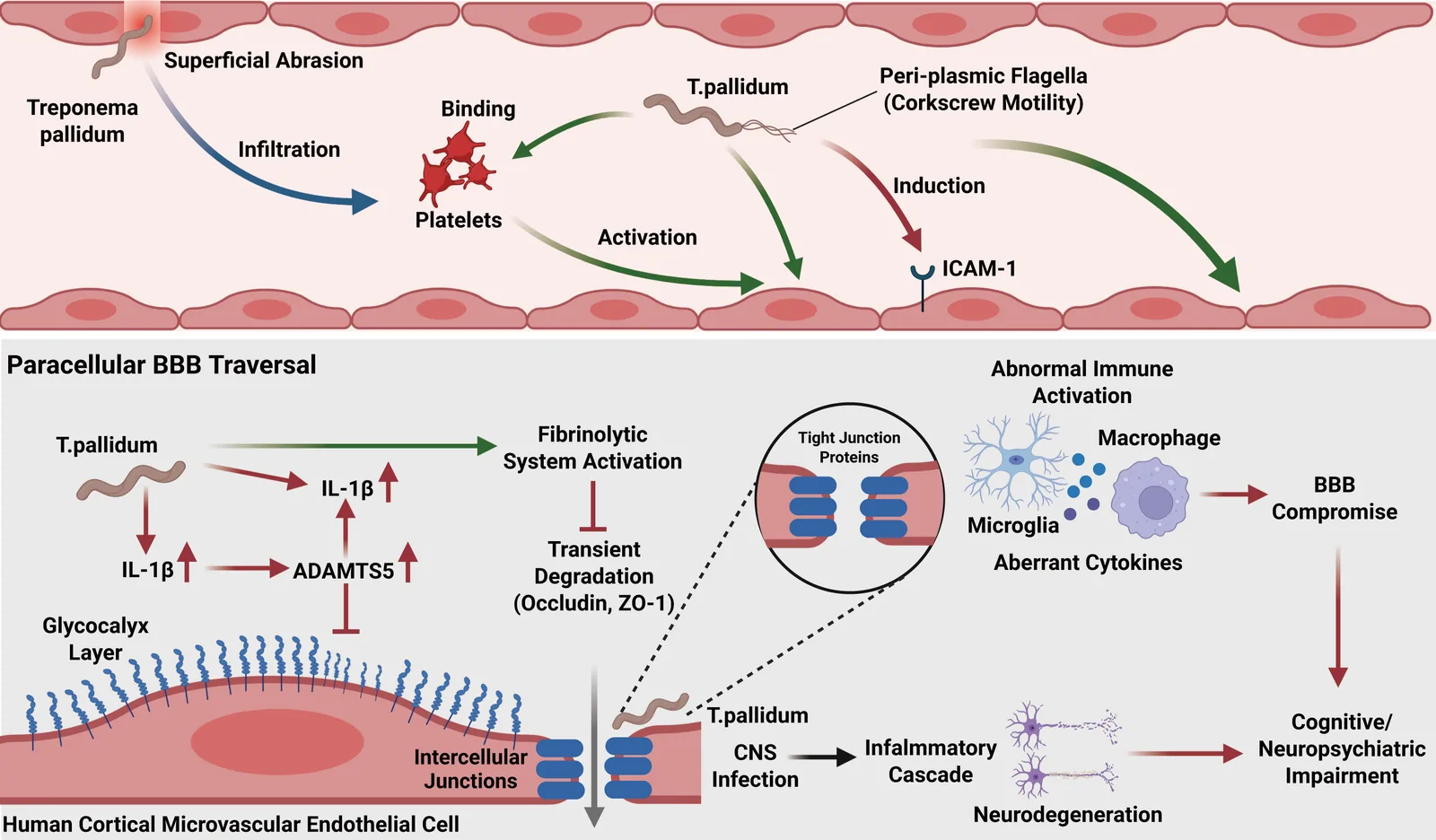
The biomechanical and molecular cascade of Treponema pallidum neuroinvasion. This schematic illustrates the early transition of Treponema pallidum from systemic circulation to the CNS. The process initiates with the spirochete’s periplasmic flagella generating corkscrew-like propulsion to navigate high-viscosity host environments, followed by vascular docking mediated by platelet binding and the induction of endothelial adhesion molecules like ICAM-1. The subsequent paracellular breach of the BBB is orchestrated through a localized molecular cascade, wherein pathogen-induced IL-1β triggers the upregulation of ADAMTS5, leading to the targeted degradation of the protective endothelial glycocalyx layer. In coordination with fibrinolytic system activation, this signaling axis facilitates the transient disassembly of tight junction proteins (e.g., Occludin, ZO-1) and increases vascular permeability, allowing the spirochete to bypass the neurovascular unit and sequester within immune-privileged CNS niches. Once established, the pathogen triggers abnormal activation of microglia and macrophages, initiating a chronic neuro-inflammatory cascade that culminates in irreversible synaptic loss, neural atrophy, and progressive cognitive or neuropsychiatric impairment.

### 4.2 Degradation of the glycocalyx and junctional proteins

The spread of *Treponema pallidum* into the CNS is a critical step in the development of neurosyphilis. To get there, the spirochete must find a way to cross the tough blood-brain barrier (BBB) (65). This invasion uses a smart, multi-part strategy. It starts by creating a pro-inflammatory microenvironment within the brain’s blood vessels. A major factor here is the production of interleukin-1β (IL-1β), which the pathogen triggers. This cytokine is strongly associated with the upregulation of a disintegrin and metalloproteinase with thrombospondin type 1 motif 5 (ADAMTS5) (**Figure 2**) (66). This specific enzyme potentially contributes to increased BBB permeability. It targets and reduces the protective glycocalyx layer on the surface of human cortical microvascular endothelial cells (hCMEC/D3) (66). By breaking down this primary physical and chemical shield, the pathogen weakens the first line of defense in specialized CNS tissues (65). The success of this breach also depends on molecular effectors like Tp0751. This protein helps the bacteria stick to and penetrate vessels via the 67-kDa laminin receptor. Additionally, TP0768 encourages monocytic cell adhesion to endothelial cells, further aiding the process (**Table 1**).

To physically enter the brain parenchyma, *Treponema pallidum* uses paracellular penetration. This is a specialized way of moving through the intercellular junctions between endothelial cells (**Figure 2**). Evidence suggests the spirochete hijacks the host’s own fibrinolytic system to briefly break down key tight-junction proteins, such as Occludin and ZO-1. This trick allows for efficient transit without causing immediate or permanent damage to the structural health of the endothelial cells themselves (67). The pathogen’s strong invasive potential and advanced immune evasion properties further support this process, helping it infiltrate the CNS quickly while avoiding early clearance by the host (68). For example, the TprK protein undergoes high-level antigenic variation to dodge antibody binding, while the adhesin TP0954 helps the pathogen bind to distal neural pathways (**Table 1**). The breach of the BBB gets worse because of abnormal cytokine expression and the irregular activation of immune cells like microglia and macrophages, which further weaken the barrier’s integrity (65). Once the spirochete crosses this specialized tissue interface and settles in the CNS, it triggers a chronic inflammatory cascade (68). This lasting immune response leads to progressive neurodegeneration, neuronal injury, and synaptic dysfunction. Ultimately, these issues result in the severe cognitive and neuropsychiatric impairments typical of neurosyphilis-induced dementia (**Figure 2**) (33, 69). Importantly, while the IL-1β/ADAMTS5 pathway represents a significant pathological axis, these early molecular events likely function within a broader, integrated network of neuroinflammatory and metabolic responses. Currently, the direct causal link between these early molecular alterations and late-stage cognitive decline remains a plausible hypothesis supported primarily by clinical correlations. Definitive functional validation and a clearer chronological mapping of this trajectory require further rigorous clarification in longitudinal studies.

## 5 CNS immune modulation, chronic neurovascular injury, and neural atrophy

### 5.1 Systemic dynamics and transition to CNS immune modulation

Generalized lymphadenopathy in syphilis is a key clinical sign that the host is trying to start a systemic immune response. However, it also shows a clear failure to actually kill off the pathogen (75). This widespread lymphatic involvement happens because *Treponema pallidum* uses the lymphatics to spread quickly through the body. This process makes the infection mainly one of the lymphoid tissues (76). During this stage, the spirochetes turn lymph nodes into persistent reservoirs. Here, the bacteria can stay indefinitely, regardless of whether visible syphilitic lesions are active or have healed (**Figure 3**) (76). Active treponemata survive for a long time in these tissues. In fact, this is a core feature of the infection that lets the disease settle in within just 48 hours of inoculation (76). While this systemic lymphadenopathy—mostly seen in the secondary stage—shows the body is trying to clear the infection, the resulting chronic state is driven by an inadequate cellular immune response. The host simply fails to clear the invasive pathogen (**Figure 3**) (75, 77). This failure is marked by poor T lymphocyte activation. Additionally, the pathogen is very good at suppressing effector cells like macrophages and dendritic cells (77). These evasion tactics include the low immunogenicity of surface antigens and the invasion of immune-privileged sites. Together, they let *Treponema pallidum* bypass host surveillance and stay persistent (42, 77). Since the host cannot fully wipe out the pathogen, the constant presence of spirochetes is associated with chronic inflammation. This eventually leads to tertiary syphilis (42). This persistent peripheral priming effectively sets the stage for a broader CNS immune modulation, creating localized ‘immune-evasive niches’ that allow the pathogen to chronically tolerate the neural parenchyma (**Figure 3**). At this late stage, the lack of control results in destructive cardiovascular and neurologic sequelae, including aortitis, dementia, and progressive neural atrophy (**Figure 3**) (75, 78).

**Figure 3:**
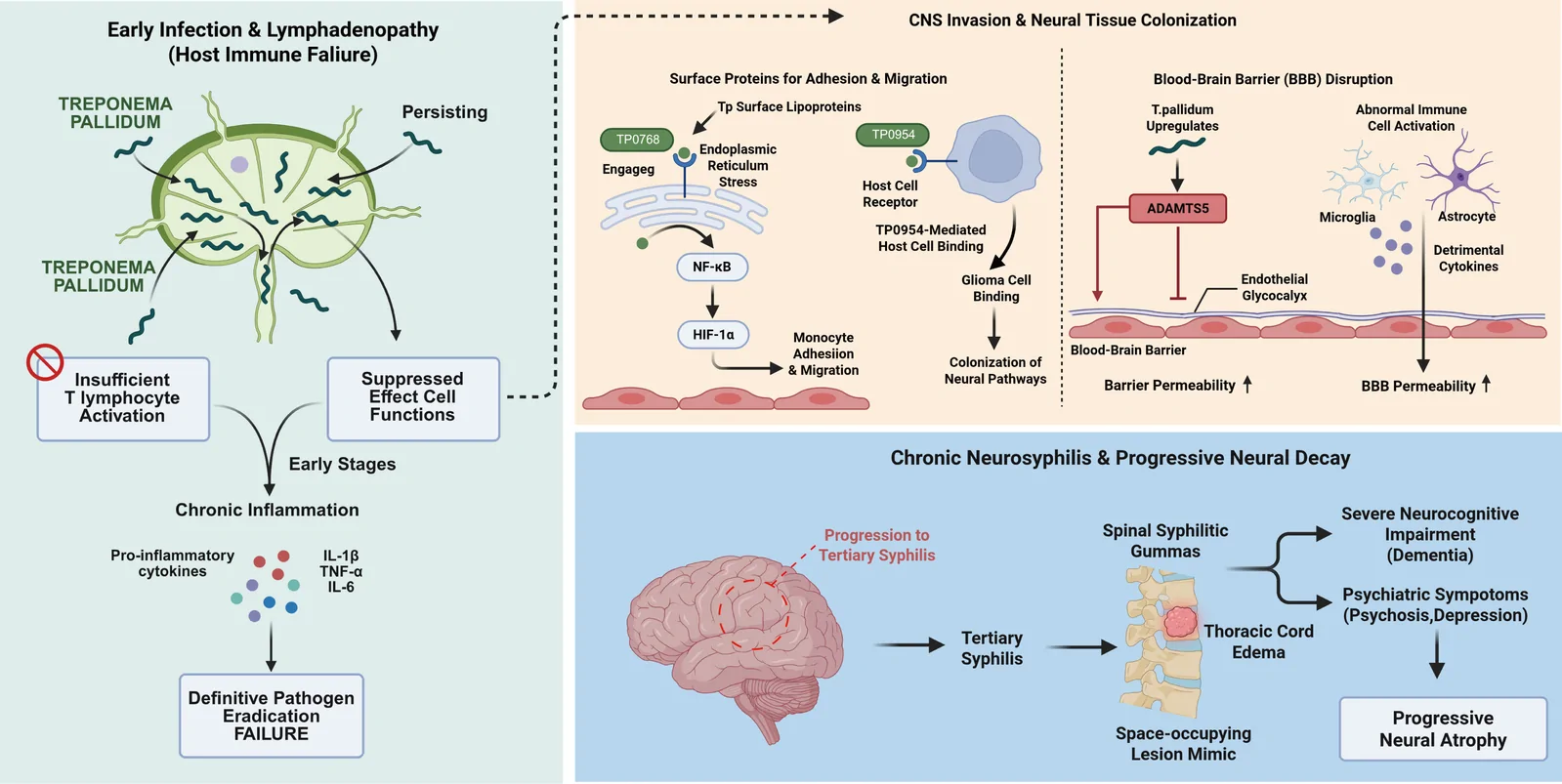
Cytotoxic signaling pathways leading to progressive neurodegenerative decay. The transition from early infection to destructive tertiary syphilis is marked by a failure of definitive pathogen eradication and the establishment of chronic inflammatory cascades. In the early stages, Treponema pallidum utilizes the lymphatic system as a persistent reservoir, where suppressed effector cell functions and insufficient T-lymphocyte activation prevent clearance. Within the CNS, specialized surface proteins—including the adhesin TP0954 and the ER-stress-inducing lipoprotein TP0768—facilitate tissue colonization and the activation of detrimental pathways such as NF-κB/HIF-1α. Chronic disruption of the BBB, exacerbated by ADAMTS5 upregulation and endothelial glycocalyx degradation, creates a persistent immune-mediated microenvironment. This long-term neuro-inflammatory state ultimately culminates in irreversible synaptic loss, manifesting clinically as dementia, psychosis, and severe neural atrophy.

### 5.2 Molecular mechanisms of chronic neurovascular injury and neural atrophy

*Treponema pallidum* can invade the CNS at any stage of infection. If the host immune system does not clear the pathogen quickly, it can lead to various debilitating forms of neurosyphilis (79). Although classical presentations like tabes dorsalis and general paresis are rare today, the disease often shows up through atypical and subtle signs. This has earned it the nickname “the great imitator” (80). Clinical symptoms often include severe neurocognitive impairment, such as dementia. Patients may also face psychiatric symptoms like psychosis, delirium, depression, mania, and personality changes (81). In some instances, neurosyphilis can even look like stroke-like syndromes or movement disorders. It can even mimic rare conditions like Capgras syndrome and Geschwind syndrome (80, 81). Some cases have even shown MRI findings that look like herpes simplex encephalitis (80). The growth of spinal syphilitic gummas, while extremely rare, is a destructive form of the disease. These can cause thoracic cord edema and act like space-occupying lesions or transverse myelitis, often starting as numbness or lower back pain (80, 82). Ultimately, the mix of these molecular and inflammatory processes causes progressive neural atrophy. This leads to the wide range of neuropsychiatric decay seen in chronic patients (**Figure 3**) (79).

The movement of spirochetes into these sensitive neural tissues is may be facilitated by specific surface lipoproteins that manage adhesion and migration through host barriers. For instance, the surface lipoprotein TP0768 promotes the migration and adhesion of monocytic cells to vascular endothelial cells (71). This process works by inducing ER stress—specifically through the protein kinase R-like endoplasmic reticulum kinase (PERK) and inositol-requiring enzyme 1 alpha (IRE-1α) pathways—and activating the NF-κB/HIF-1α signaling pathway (71). Such activation increases the expression of inflammatory markers like ICAM-1, MCP-1, and IL-8, which significantly boosts the adhesion of immune cells to the endothelium (71). Additionally, TP0954 serves as a critical surface adhesin that allows *Treponema pallidum* to bind to various host cells, including placental tissues and glioma cell lines (73). By binding to heparan sulfate and dermatan sulfate within the ECM, TP0954 helps the pathogen disseminate and colonize distal neural pathways (73). Notably, the ability of this protein to bind human placental sections suggests it plays a major role in placental colonization and potential transplacental transmission, mirroring how the pathogen settles into sensitive CNS tissues (**Figure 3**) (73).

Beyond direct adhesion, the pathogen disrupts the BBB by upregulating specific proteins that increase barrier permeability. Specifically, *Treponema pallidum* triggers the expression of ADAMTS5 in brain microvascular endothelial cells, which leads to a significant reduction in the protective endothelial glycocalyx on the cell surface (**Figure 3**) (66). This breach is further exacerbated by the abnormal activation of immune cells and the release of detrimental cytokines, such as interleukin-1β. This signaling further facilitates ADAMTS5 expression and compromises BBB integrity (65, 66). Furthermore, *Treponema pallidum* utilizes its unique cell structure—specifically the low abundance of surface transmembrane proteins and the lack of LPSs—to effectively conceal itself from the host immune system (83). This “stealth” strategy allows for sustained tissue invasion and long-term persistence within the CNS parenchyma by avoiding robust immune detection (83). The chronic inflammation resulting from this persistence involves the disruption of intercellular junctions and endothelial cell injury, which are key factors in the spread of infection (65, 71). Consequently, the failure of definitive pathogen eradication results in the progressive neural atrophy and cognitive abnormalities characteristic of late-stage neurosyphilis (65, 79).

## 6 Current approaches and advanced methodologies

### 6.1 Advancements in cultivation

The historical study of *Treponema pallidum* subsp. *Pallidum* (TPA), the causative agent of syphilis, was fundamentally obstructed for over a century by the inability to sustain the organism in a continuous laboratory culture (18, 20). For decades, researchers had to rely almost exclusively on rabbit orchitis models for propagation. This necessity imposed severe limitations on the field due to the high costs, ethical concerns, and technical complexities inherent in animal-based research (**Table 2**) (18, 84). This reliance on *in vivo* maintenance meant that *Treponema pallidum* remained one of the few major human bacterial pathogens that could not be manipulated through standard microbiological techniques (20). However, a transformative breakthrough arrived with the development of a microaerobic tissue culture system that finally enabled long-term, continuous *in vitro* growth (20). This system utilizes a specialized medium known as *Treponema pallidum* Culture Medium 2 (TpCM-2) within a co-culture environment using the Sf1Ep rabbit epithelial cell line (18, 20). The success of this methodology depends on a precise microaerobic atmosphere and specific nutrient requirements. Key factors include the addition of 20% fetal bovine serum and complex basal media like CMRL 1066 or M199, which provide the essential nutrients missing from simpler formulations (**Figure 4**; **Table 2**) (18).

**Table 2:** Evolution of experimental platforms: from animal models to genetic engineering.

Research platform	Core methodology / tools	Technical breakthroughs & advantages	Key references
In Vivo Models	Rabbit Orchitis Model.	Maintained wild-type infectivity and morphology for over a century.	(76, 98)
In Vitro Culture	Sf1Ep-TpCM-2 Microaerobic System.	Supported continuous growth for >3 years with 94% proteome coverage.	(20, 99, 100)
Genetic Tools	Suicide Vectors & CRISPR/Cas9.	Enabled investigation of virulence factors and precise DNA modification.	(92, 101)
Real-time Imaging	GFP-expression & AIEgen Labeling.	Spatiotemporal tracking of BBB crossing and immune clearance dynamics.	(59, 87)

This table highlights how the transition to in vitro systems has overcome historical bottlenecks in syphilis research.

**Figure 4:**
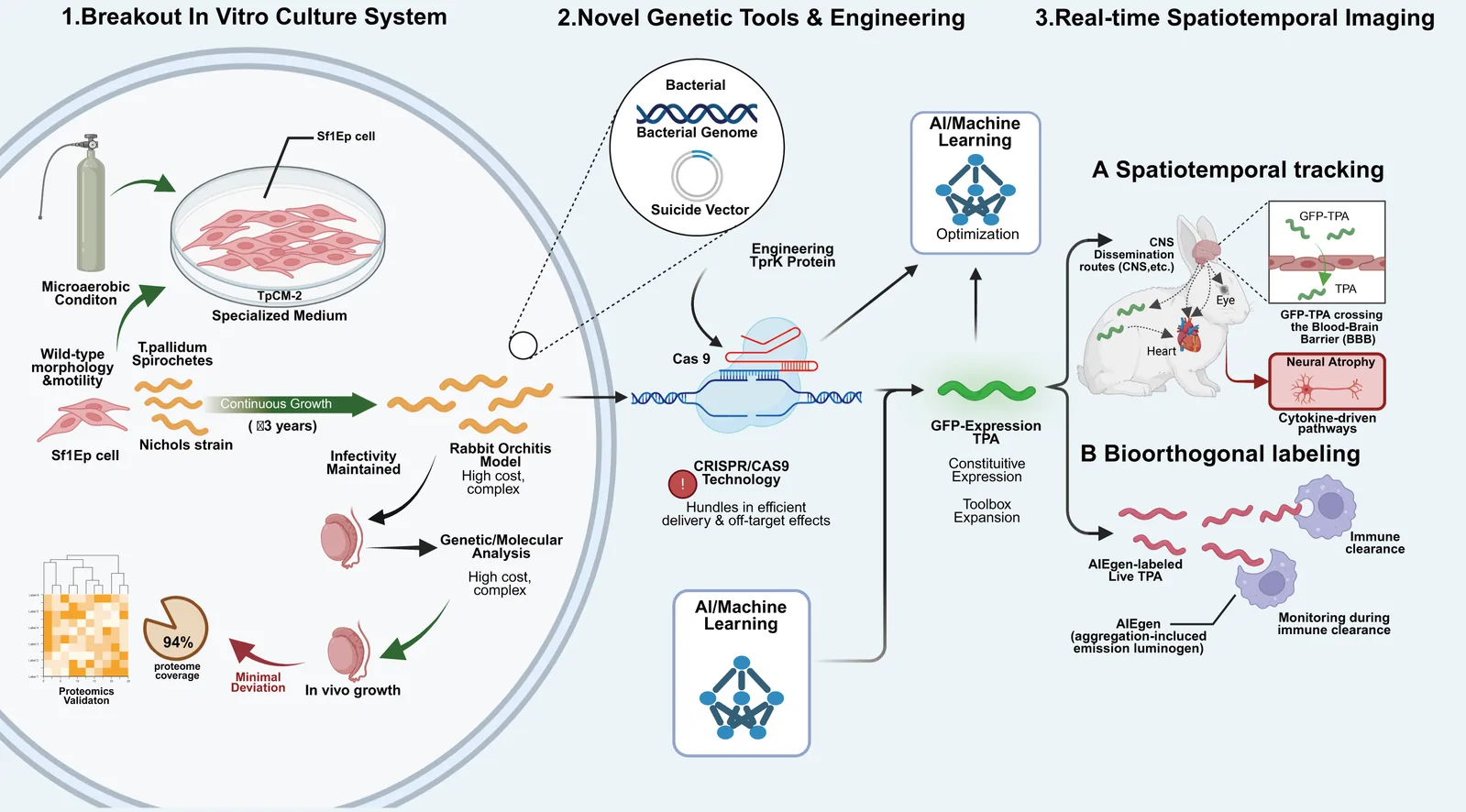
Breakthroughs in in vitro cultivation, genetic engineering, and real-time tracking. Recent technological advancements have overcome historical barriers in syphilis research, shifting focus from costly rabbit models to high-resolution molecular analysis. The development of the Sf1Ep-TpCM-2 microaerobic tissue culture system now supports continuous logarithmic growth of Treponema pallidum for over three years while maintaining 94% proteome coverage and wild-type infectivity. These culture breakthroughs enable the application of novel genetic tools, such as suicide vector-mediated engineering and CRISPR/Cas9, despite existing delivery challenges. Spatiotemporal tracking is further enhanced by the engineering of GFP-expressing strains and catalyst-free bioorthogonal labeling using AIEgens, which allow for the real-time visualization of spirochete dissemination and immune clearance dynamics within the host.

This cultivation system has proven remarkably robust, supporting the continuous growth of the Nichols strain and various clinical isolates, such as SS14 and Mexico A, for periods now exceeding three years (18, 20). Recent comparative analysis of seven such strains has further quantified their physiological diversity, revealing significant growth rate differences between Nichols-like and SS14-like clusters, with generation times ranging from approximately 33 to 44 hours (85). Throughout this extended *in vitro* passage, the treponemes exhibit a typical bacterial growth curve consisting of logarithmic and stationary phases. Notably, they maintain their wild-type spiral morphology and characteristic motility (18, 20). Crucially, the bacteria preserved their biological integrity and virulence, showing full retention of rabbit infectivity even after prolonged periods away from a host environment (18, 20). The Sf1Ep cells play a vital role in this process; they are required for the sustained multiplication and movement of the spirochetes. However, the system requires careful titration of initial cell numbers to avoid reducing bacterial survival (18). By providing reliable protocols for both standard laboratory environments and simplified setups like Brewer jars, this breakthrough has democratized syphilis research. It allows for more consistent and accessible investigation into the pathogen’s biology (**Figure 4**) (18).

Beyond the mere maintenance of the bacteria, this long-term culture system has opened the door to advanced genetic and molecular exploration that was once impossible. Specifically, the ability to perform limiting dilution experiments has allowed researchers to establish clonal populations from as few as 0.5 organisms per well. This provides a critical tool for studying microevolution and genetic heterogeneity within strains like Nichols (86). For instance, longitudinal deep sequencing of *in vitro*-propagated treponemes has revealed that antigenic variation in the TprK protein occurs through segmental gene conversion even in the complete absence of host immune pressure, providing new insights into the baseline mechanisms of immune evasion (44). Building on this platform, new resources have emerged to enhance visualization. These include the engineering of genetically encoded fluorescent tags for both *Treponema pallidum* and Sf1Ep cells, allowing scientists to better capture host-pathogen interactions (**Figure 4**; **Table 2**) (87). Furthermore, the validity of this *in vitro* approach as a surrogate for animal models has been confirmed through comprehensive molecular profiling. Comparative transcriptomic analyses have shown that the gene expression patterns of *Treponema pallidum in vitro* are remarkably similar to those during rabbit infection, with over 90% of genes exhibiting concordant transcript levels (88). Complementing this, recent mass spectrometry analyses revealed that *in vitro*-cultured treponemes achieve 94% proteome coverage, showing only minimal deviations from the protein expression profiles of *in vivo*-grown organisms (**Table 2**) (84). These findings reinforce that the Sf1Ep-based system is a robust alternative to rabbit models. In essence, it facilitates deep dives into the mechanisms of immune evasion, antibiotic efficacy, and the overall pathogenesis of syphilis (84, 87).

### 6.2 Genetic tools and imaging

The pathogenesis of TPA is involves a diverse array of functional proteins that facilitate host cell adhesion, tissue invasion, and immune evasion, making them primary targets for diagnostic and therapeutic innovation (89). However, the study of these critical proteins has historically been hindered by its fastidious nature, characterized by a slow doubling time and a strict requirement for complex, host-cell-integrated microaerobic environments (43). For decades, the lack of robust genetic manipulation systems meant that researchers could only observe the pathogen’s effects rather than probe its molecular foundations (43). However, recent breakthroughs in long-term *in vitro* cultivation—using specialized media and Sf1Ep rabbit epithelial cells—have catalyzed the development of primary genetic tools (87). This breakthrough further enabled high-quality transcriptome profiling, which experimentally validated previously hypothetical genes and revealed complex transcription units, thereby providing a precise molecular map for functional genomics (90). Specifically, the successful engineering of TPA strains using suicide vectors to investigate critical virulence factors has marked a new era in syphilis research (43). For instance, researchers have utilized these suicide vectors to create loss-of-function mutants, such as strains with an artificially impaired TprK antigenic variation system (43). By eliminating approximately 96% of the chromosomal donor cassettes for the TprK protein, studies have demonstrated that while these mutants maintain identical growth rates *in vitro*, they exhibit significant attenuation and reduced treponemal burden in rabbit models (43) (Whitehead, 2023). This confirms that genetic manipulation can now directly elucidate how specific proteins facilitate immune avoidance and persistent infection (**Figure 4**; **Table 2**) (43).

Despite these advancements, the transition to more sophisticated systems like CRISPR/Cas9 technology remains fraught with challenges (91). Recently, the first successful genetic manipulation of *Treponema pallidum* was achieved through homologous recombination using a suicide vector to replace the tprA gene, proving that the spirochete is genetically tractable (92). However, while CRISPR/Cas9 offers revolutionary potential for precise DNA modifications and novel therapeutic strategies, its application to TPA is currently hindered by significant technical hurdles. These limitations are often echoed in broader genomic research, particularly regarding the inherent inefficiencies of current molecular tools (93). In other complex biological systems, these hurdles often stem from the large size of the Cas9 enzyme and the limited packaging capacity of viral vectors, which necessitates inefficient multi-vector systems (94). These include the lack of efficient shuttle vectors (87). Difficulties in achieving high-efficiency cellular delivery through the spirochete’s complex membrane structure also remain a primary concern (91). Furthermore, the risk of off-target effects and potential immune responses to the Cas9 machinery itself must be addressed to ensure the accuracy of genomic editing in such a selective pathogen (91). Emerging opportunities to overcome these limitations lie in the integration of interdisciplinary technologies, such as artificial intelligence (AI) and machine learning (91). Recent frameworks suggest that such computational strategies are essential for evolving CRISPR into a more reliable and scalable platform (93). These computational advancements present a pathway to refine the precision of gene editing by predicting optimal guide RNA sequences and minimizing off-target interactions (91). In essence, such AI-driven optimization may eventually allow for the same level of precise genetic control in TPA that is currently available for more easily cultured bacterial models where gene disruption and tagging techniques are routine (**Figure 4**; **Table 2**) (91, 95).

Parallel to the development of editing tools, the engineering of infectious TPA strains that constitutively express fluorescent tags has significantly expanded the syphilis research toolbox (96). Recent studies have successfully integrated green fluorescent protein (GFP) into the TPA Nichols strain, producing “GFP+ TPA” that exhibits wild-type morphology, motility, and infectivity (96). These fluorescent strains enable high-resolution visualization of spirochete-host interactions in real-time. This ranges from *in vitro* co-cultivation with host cells to tracking dissemination within complex organ systems (**Table 2**) (96). Beyond genetic encoding, researchers have developed red-shifted GFP and blue fluorescent protein (BFP) tags for Sf1Ep cells to better capture the physical interaction between the host and pathogen (87). Furthermore, advanced catalyst-free bioorthogonal labeling strategies using aggregation-induced emission luminogens (AIEgens) allow for the monitoring of live spirochetes during immune clearance without affecting their biological activity (97). These imaging modalities are particularly vital for elucidating how TPA disseminates to the CNS by breaching the BBB (96). Understanding the interaction between TPA and the BBB is essential for revealing neurosyphilis pathogenesis (65). By combining fluorescent tracking with an understanding of cytokine-driven pathways that disrupt endothelial junctions, researchers can finally visualize the spatiotemporal progression of neural atrophy (**Figure 4**) (65).

## 7 Diagnostics, treatment and neurological prognosis

### 7.1 Diagnostic challenges and nursing standards

The clinical management of neurosyphilis is increasingly complicated by persistent diagnostic challenges. Specifically, there is a critical need for advanced point-of-care assays to differentiate between active neuro-invasion and past CNS involvement (102, 103). Current diagnostic protocols remain complex and non-standardized. They typically require a meticulous synthesis of heterogeneous clinical symptoms, positive serum treponemal and non-treponemal tests, and detailed cerebrospinal fluid (CSF) analysis to confirm active disease (**Figure 5**) (104, 105). While the CSF Venereal Disease Research Laboratory (VDRL) test is often regarded as a mainstay, its low sensitivity frequently necessitates the integration of multiple serologic tests. This can significantly delay turnaround times and hinder timely clinical decision-making (106, 107). This diagnostic ambiguity is particularly pronounced in the 21st-century epidemic, where the “great imitator” manifests through a diverse array of neuropsychiatric disorders and focal neurologic signs that can easily be misidentified as other conditions (103). Consequently, research highlights a vital data gap and an urgent need for novel point-of-care diagnostics that can overcome existing technological and structural barriers. Such tools are essential for the accurate identification of active infections and appropriate patient management (102, 107).

**Figure 5:**
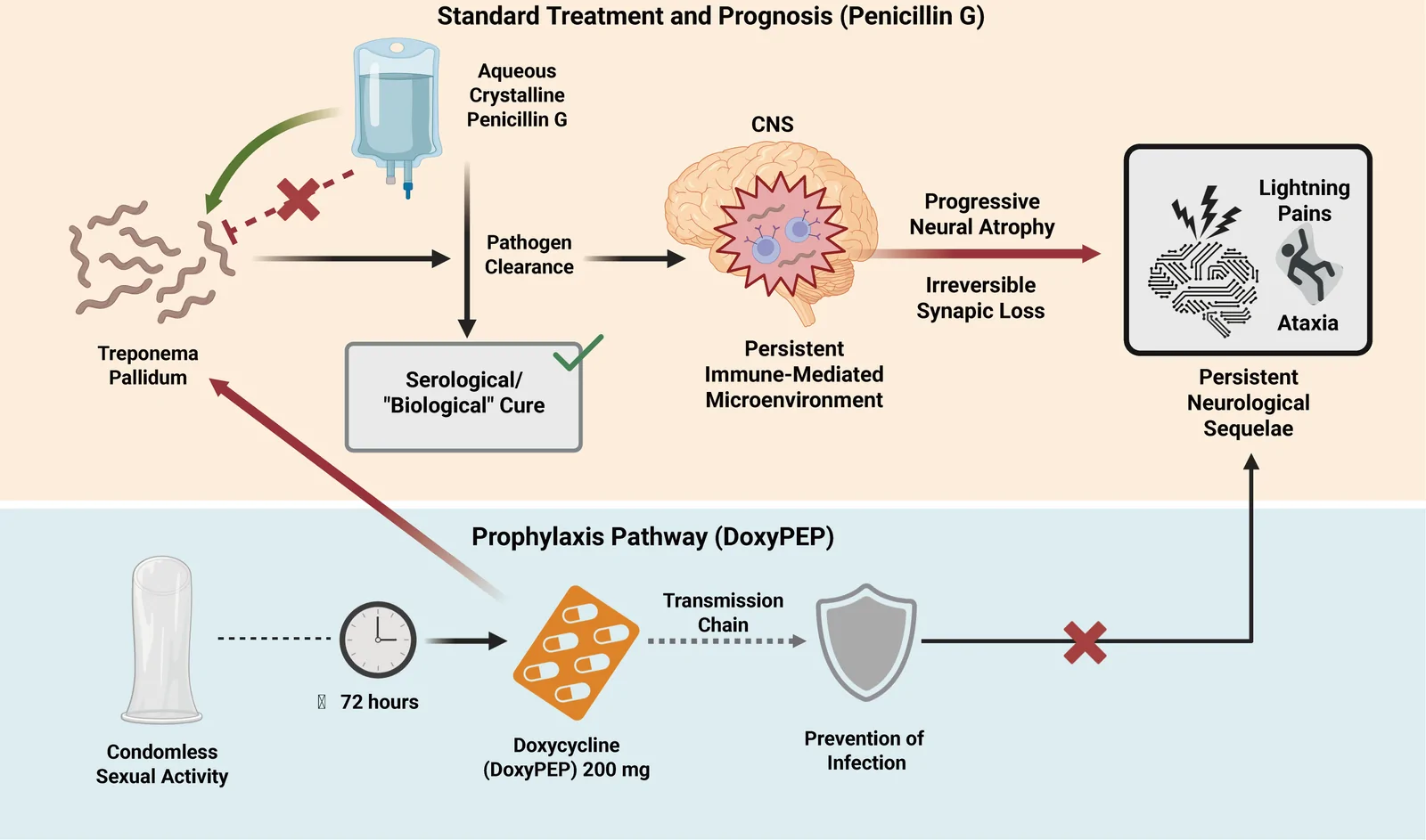
Current therapeutic standards, neurological prognosis, and prophylaxis models. This figure delineates the clinical dichotomy between serological cure and functional recovery. While aqueous crystalline penicillin G remains the gold standard for achieving serological cure, it is frequently insufficient to arrest the established neuro-inflammatory cascade, leading to permanent neurological sequelae such as “burnt-out” tabes dorsalis and intractable ataxia. To address the global resurgence of the pathogen, modern prophylactic strategies have pivoted toward DoxyPEP. Administering 200 mg of doxycycline within 72 hours of condomless sexual activity has demonstrated significant efficacy in reducing infection incidence among high-risk populations. This pharmaceutical layer aims to disrupt the transmission chain before the spirochete can establish a suppressive chemotactic microenvironment within the host.

Regarding the standard of care, the medical community continues to rely almost exclusively on aqueous crystalline penicillin G as the gold standard for eradicating *Treponema pallidum* from the CNS (**Figure 5**) (79, 105). However, this continued reliance faces significant hurdles. These include recurring global shortages of benzathine penicillin G, logistical distribution issues, and the emergence of antibiotic-resistant strains, which necessitate the use of secondary treatments like doxycycline (107, 108). A more profound challenge lies in managing late-stage, irreversible neurological damage. Even successful pathogen clearance and a “serological cure” through penicillin therapy cannot reverse advanced synaptic and tissue loss (**Figure 5**) (79, 104). Autopsy findings in treated patients have revealed mixed brain pathology, including advanced vascular pathology and atrophy of the frontotemporal areas. This demonstrates that the “post-treatment” phase is often characterized by permanent complications such as tabes dorsalis, general paresis, or progressive dementia (105). Thus, modern nursing and medical standards must shift toward a multidisciplinary approach. In essence, while penicillin effectively halts the infection, it is often insufficient to restore functional recovery once structural neurological damage has already occurred (**Figure 5**) (104, 108).

### 7.2 Clinical management and therapeutic strategies

To provide optimal clinical management for *Treponema pallidum* infections, understanding the exact treatment regimens and correct dosages is paramount (**Table 3**). The gold standard for treating neurosyphilis is aqueous crystalline penicillin G, administered intravenously at a dosage of 18–24 million units per day, divided as 3–4 million units every 4 hours or given via continuous infusion, for a total duration of 10–14 days (109, 110). Conversely, benzathine penicillin G serves as the primary regimen for non-neurological manifestations. For early syphilis (primary, secondary, or early latent), a single intramuscular injection of 2.4 million units is recommended. For late latent syphilis or cases of unknown duration, the standard regimen requires three intramuscular injections of 2.4 million units each, spaced one week apart, achieving a total dose of 7.2 million units (111, 112).

**Table 3:** Therapeutic regimens and clinical management of *Treponema pallidum* infections.

Antibiotic regimens	Clinical manifestations & target population	Route & dosage	Key clinical considerations	Key references
Aqueous crystalline penicillin G	Neurosyphilis	3–4 million units every 4 hours (or via continuous infusion to reach 18–24 million units/day), intravenously (IV), for 10–14 days.	Irreplaceable due to excellent blood-brain barrier penetration. In case of confirmed IgE-mediated allergy, mandatory desensitization under strict monitoring is required.	(109, 110)
Benzathine penicillin G	Early Syphilis. Late Latent Syphilis.	Early: 2.4 million units in a single dose, intramuscularly (IM). Late Latent: 2.4 million units once weekly, intramuscularly (IM), for 3 consecutive weeks (total: 7.2 million units).	Primary regimen for non neurological manifestations. Highly effective in treating maternal infection and preventing vertical transmission. Requires strict weekly spacing for late-stage latent infection.	(111, 112)
Doxycycline	Early Syphilis. Latent Syphilis.	Early: 100 mg twice daily, orally, for 14 days. Latent: 100 mg twice daily, orally, for 28 days.	Serves as an effective alternative for patients with confirmed penicillin allergy. Requires strict adherence to the extended 28-day regimen in latent stages to ensure clearance.	(108, 115)
Tetracycline	Early Syphilis. Latent Syphilis.	Early: 500 mg four times daily, orally, for 14 days. Latent: 500 mg four times daily, orally, for 28 days.	Serves as another effective oral alternative for penicillin-allergic patients. Multiple daily dosing (four times daily) may impact patient compliance compared to doxycycline.	(108, 115)
Ceftriaxone	Neurosyphilis. Maternal Infection & Congenital Syphilis Prevention.	1–2 g once daily, intravenously (IV) or intramuscularly (IM), for 10–14 days.	Particularly recommended as an effective alternative approach for neurosyphilis. Documented as a successful alternative to cure maternal infection and achieve prevention of congenital syphilis when severe non-IgE-mediated reactions (e.g., SJS) make penicillin desensitization impossible.	(108, 115)

This table outlines the current gold-standard antibiotic regimens, clinical dosages, and key considerations for the management of different syphilitic stages and neuro-invasion.

However, the clinical implementation of these standard penicillin regimens can be severely challenged by patient hypersensitivity, requiring comprehensive assessment and alternative therapeutic strategies. When managing suspected penicillin allergy, clinicians must first obtain a detailed allergy history and perform penicillin skin testing to confirm true, IgE-mediated type I hypersensitivity (113). Clinically significant hypersensitivity is uncommon, but accurate evaluation prevents the unnecessary use of suboptimal broad-spectrum alternatives (113). For pregnant women and individuals with neurosyphilis, penicillin remains the irreplaceable gold standard due to its unmatched efficacy and capacity to cross the placental and blood-brain barriers (109, 114). Consequently, when a severe allergy is confirmed in these high-risk populations, the optimal clinical decision is to perform mandatory penicillin desensitization under strict medical monitoring, followed by immediate standard penicillin therapy (109, 114). For patients with penicillin allergy, oral doxycycline (100 mg twice daily) or tetracycline (500 mg four times daily) for 14 days in early syphilis and 28 days in latent syphilis serve as effective alternatives (**Table 3**) (108, 115). Ceftriaxone (1–2 g daily, intravenously or intramuscularly for 10–14 days) is also recommended, particularly for neurosyphilis (108, 115). However, azithromycin is no longer preferred due to widespread resistance mutations (108).

Special clinical attention must also be dedicated to specific high-risk scenarios, particularly gestational infections and acute systemic post-treatment reactions. Gestational syphilis poses severe fetal risks, leading to adverse pregnancy outcomes such as abortion, stillbirth, prematurity, low birth weight, and neonatal mortality, alongside congenital syphilis malformations (116–118). Administering appropriate benzathine penicillin G therapy based on the maternal infection stage is highly effective in treating maternal infection, crossing the placental barrier, and preventing vertical transmission (117–119). Erythromycin cannot be relied upon because it fails to cross the placental barrier effectively to treat the fetus (118). For pregnant women with confirmed penicillin hypersensitivity, executing an oral or intravenous penicillin desensitization protocol under strict medical surveillance remains a standard and highly successful strategy to achieve safe cure (119). However, when severe non-IgE-mediated reactions like Stevens-Johnson syndrome make penicillin desensitization unsafe or impossible, alternative regimens must be considered (120). In such cases, ceftriaxone has been documented as an effective alternative approach that successfully cures maternal infection and achieves the prevention of congenital syphilis (120). In addition to managing gestational risks, clinicians must anticipate the immunological consequences of treatment, as following standard antibiotic therapy such as penicillin, the sudden destruction of *Treponema pallidum* triggers a prominent systemic inflammation known as the Jarisch-Herxheimer reaction. This reaction occurs during early antibiotic treatment, but its clinical severity shows no definitive correlation with changes or levels of circulating immune complexes. Instead, the pathogenesis is driven by key functional proteins and antigens of *Treponema pallidum* that promote strong inflammatory responses and tissue damage (89, 121).

### 7.3 Prophylactic strategies

To mitigate the escalating incidence of syphilis and its potential progression into the debilitating stages of neurosyphilis, modern prophylactic strategies have pivoted toward the adaptation of Pre-exposure Prophylaxis (PrEP) and Post-exposure Prophylaxis (PEP) models. Specifically, the use of doxycycline post-exposure prophylaxis (DoxyPEP) has emerged as a cornerstone in reducing the risk of bacterial sexually transmitted infections (STIs). This typically involves a single 200 mg dose of doxycycline administered within a 72-hour window following condomless sexual activity (**Figure 5**) (122). Clinical guidelines, including those recently released by the CDC, recommend this regimen primarily for men who have sex with men (MSM) and transgender women (TGW) who have been diagnosed with a bacterial STI in the previous 12 months (123). Extensive clinical trials have demonstrated that this intervention can significantly reduce syphilis and chlamydia infections by over 70%, while providing a moderate reduction in gonococcal infections by approximately 50% (123). By providing a rapid pharmaceutical intervention, DoxyPEP serves to disrupt the transmission chain before the pathogen can establish a suppressive chemotactic microenvironment within the host. Consequently, it is hypothesized that DoxyPEP might mitigate the risk of early neuro-invasion and prevent the establishment of CNS sanctuaries; however, direct clinical evidence confirming its efficacy in preventing CNS involvement remains limited and warrants cautious interpretation (**Figure 5**) (124).

The integration of these prophylactic measures is backed by sophisticated agent-based modeling and longitudinal efficacy studies focusing on urban high-risk populations (125). Research suggests that DoxyPEP is especially effective among gay, bisexual, and other men who have sex with men (GBMSM) and TGW, who remain disproportionately affected by the ongoing STI epidemic (126). Notably, modeling data from urban centers like Philadelphia indicate that even a modest uptake of 20% within these communities—when paired with high adherence—could reduce the cumulative incidence of syphilis infections by approximately 10% over a decade (125). Furthermore, the evolution of these strategies has expanded to explore doxycycline-based Pre-exposure Prophylaxis (Doxy-PrEP) (127). While PrEP has historically been synonymous with HIV prevention via antiretrovirals (128), researchers are now investigating daily doxycycline as a proactive way to prevent the initial acquisition of bacterial pathogens in sexual minority populations (127). This transition from reactive treatment to proactive prevention represents a vital shift in managing the health of at-risk groups by stopping the pathogen from establishing a foothold in the first place (129).

However, the implementation of broad-spectrum antibiotic strategies remains a subject of intense clinical debate, specifically regarding the balance between individual benefit and public health risks (122). Medical consensus emphasizes that these interventions should not be viewed as population-level mandates; instead, they serve as selective tools for individuals at the highest risk (122). A primary concern is that such widespread use might accelerate antimicrobial resistance (AMR) within the human microbiome and among other bacterial pathogens, such as N. gonorrhoeae (127). Despite these concerns, DoxyPEP is increasingly recommended as a secondary prevention layer that complements routine screening, risk reduction counseling, and consistent condom use (123). While intravenous penicillin G remains the definitive standard for treating active neurosyphilis and achieving a serological cure, consensus underscores that it cannot always reverse existing neurological damage (130). Therefore, the flexibility offered by alternative tetracyclines in a prophylactic context represents a necessary evolution. In essence, it helps disrupt the infection cycle before irreversible neurological sequelae, such as ataxia or lightning pains, can manifest (**Figure 5**) (130).

## 8 Integrated pathogenic framework and evidence-level analysis

To provide a clear overview of the current evidence base, we have stratified the major mechanistic claims presented in this review according to their level of supporting evidence (**Table 4**). Mechanisms such as the low outer-membrane antigenicity and TprK-mediated antigenic variation are classified as Strong, backed by extensive ultrastructural, biochemical, and genetic studies. The IL-1β/ADAMTS5 glycocalyx degradation axis and tight-junction remodeling/EndoMT are rated as Moderate, primarily supported by *in vitro* endothelial cell models and transcriptomic data. In contrast, the formation of an “immune-evasive niches” in the CNS, the potential of DoxyPEP to prevent CNS sanctuary establishment, and the direct linear causal relationship to irreversible neurovascular injury and dementia remain at the Low evidence level, representing plausible hypotheses that require further experimental validation. This evidence stratification highlights both the solid foundations and the remaining gaps in our understanding of Treponema pallidum-induced CNS damage.

**Table 4:** Stratification of mechanistic claims based on evidence levels.

Core claims in review	Level of current evidence	Key supporting evidence & source in text	Key references
Low Outer-Membrane Antigenicity	Strong	Freeze-fracture/deep-etching analyses; biochemical confirmation of buried lipoproteins (e.g., Tp47).	(30, 33, 34)
TprK-Mediated Antigenic Variation	Strong	Long-read sequencing data; loss-of-variation phenotypes in tprK KO strains (SS14-DC).	(43, 44, 52)
IL-1β/ADAMTS5 Endothelial Glycocalyx Axis	Moderate	Human cortical microvascular endothelial cell (hCMEC/D3) models; glycocalyx degradation assays.	(65, 66)
Tight-Junction Remodeling & EndoMT	Moderate	Time-course transcriptomics;vimentin-triggered fibronectin disaggregation	(63, 131, 132)
Formation of "immune-evasive niches" in CNS	Low	Treponema pallidum enolase inducing microglia/macrophage apoptosis and blocking autophagic flux.	(42, 133)
DoxyPEP Prevention of CNS Sanctuary Establishment	Low	Extrapolation from recent DoxyPEP trials for STIs (syphilis incidence reduction data).	(123)
Linear Causal Chain to Irreversible Neurovascular Injury & Dementia	Low	Clinical endpoint correlation; post-mortem neuropathology of burnt-out tertiary cases.	(65, 69)

Strong: Fully Validated.

Moderate: In Vitro Evidence; Requires Further Validation.

Low: Speculative Hypothesis; Requires Further Validation.

This table categorizes the core pathogenic mechanisms and clinical hypotheses discussed in this review according to their current level of experimental validation and supporting evidence.

## 9 Concluding remarks and future perspectives

The global resurgence of syphilis, coupled with the emerging threat of antibiotic resistance and supply chain vulnerabilities for standard treatments like benzathine penicillin G, necessitates a paradigm shift toward identifying novel drug targets that can disrupt the sophisticated immune evasion strategies of *Treponema pallidum* (**Table 5**) (134, 135). Beyond traditional bactericidal approaches, targeting specific virulence factors such as *Treponema pallidum* enolase (Eno) is critical, as this multifunctional enzyme serves as a cornerstone for the pathogen’s survival within the host (45). Notably, Eno facilitates Treponema pallidum’s dissemination by promoting macrophage apoptosis and activating suppressive signaling pathways—specifically the ROS/NF-κB and P2X7R, and mTORC1/TFEB/autophagy axes—which allow the spirochete to bypass immune clearance (**Table 5**) (45, 133). Furthermore, focusing on the structural defenses of the “elusive corkscrew” offers a strategic opportunity; specifically, the immunodominant extracellular loops (ECLs) of outer membrane proteins, such as the FadL orthologs TP0856, TP0858, and TP0865, represent high-priority targets (**Table 5**) (39, 136). Neutralizing these ECLs can restore functional host immune responses by promoting opsonophagocytosis and inhibiting the spirochete’s inherent motility and viability (39).

**Table 5:** Integration of pathogenic mechanisms, host immune subversion, and neurodegenerative outcomes in neurosyphilis.

Pathogenic stage	Molecular drivers & signaling hubs	Host immune response & microenvironmental shift	Clinical impact & neurodegenerative sequelae	Key references
I. Initial CNS Invasion	Tp0751 (Adhesin); ADAMTS5 (Glycocalyx degradation)	Endothelial-to-mesenchymal transition; transient breakdown of Occludin/ZO-1 junctions	Early asymptomatic neuro-invasion; breach of the blood-brain barrier	(66, 131, 137)
II. Immune Evasion & Persistence	TprK (Antigenic variation); Tp92 (Apoptosis inhibition)	Creation of "Immune Cold" zones; sequestration in immune-privileged niches; delayed neutrophil response	Establishment of asymptomatic latency and chronic treponemal burden	(37, 43)
III. Chronic Neuro-inflammation	Treponema pallidum Enolase; TP0768 (ER stress inducer)	Activation of ROS/NF-κB axis; abnormal activation of microglia and astrocytes; aberrant cytokine profiles (↑IL-6, ↑IL-8)	Progressive synaptic loss and sustained neuro-inflammatory cascade	(45, 62, 65)
IV. Irreversible Neural Decay	Dysregulation of Matrix Metalloproteinases (MMPs); matrix remodeling	Chronic disruption of the neurovascular unit; permanent immune-mediated microenvironment	Neural Atrophy; Dementia; Psychosis; Permanent frontotemporal brain pathology	(78, 79)

This comprehensive table synthesizes current high-impact evidence regarding the “stealth” transition from systemic infection to irreversible neural decay.

Elucidating the intricate molecular “tug-of-war” within the neural microenvironment is paramount for averting the devastating neurological sequelae of infection, which are now recognized to occur much earlier in the disease course than previously thought (78). *Treponema pallidum* exhibits a remarkable capacity for rapid vascular dissemination, crossing the BBB to establish early CNS involvement (78). Central to this invasive process is the adhesin Tp0751, which facilitates spirochete attachment to the vascular endothelium by targeting the 67-kDa LamR—a mechanism notably shared by other potent neuroinvasive bacterial pathogens (**Table 5**) (137). Upon interaction with brain microvascular endothelial cells, *Treponema pallidum* triggers a profound transcriptional reprogramming characterized by an EndoMT and the induction of Rho GTPase signaling (**Table 5**) (131). In essence, this molecular shift induces cellular contraction and compromises vascular integrity, effectively breaking down the physiological barriers intended to protect the brain parenchyma (131, 132).

This disruption of the neural microenvironment is further exacerbated by an aberrant host immune response, where altered cytokine profiles create a permissive inflammatory milieu for BBB penetration (62). Specifically, the upregulation of pro-inflammatory and angiogenic markers such as IL-6, IL-8, and VEGF, alongside the downregulation of chemoattractants like MCP-1, promotes a state of vascular dysfunction that facilitates treponemal traversal (**Table 5**) (62). Moreover, the pathogen modulates the host’s ECM organization and innate immune signaling pathways, contributing to a suppressive chemotactic microenvironment that hinders effective spirochete clearance (65, 138). Deepening our understanding of these multifaceted interactions—from endothelial junction disruption to the induction of abnormal immune cell activation—is essential for developing targeted interventions, leveraging both molecular insights and neuroinformatics-based computational models to predict disease persistence (65, 139). In essence, only by neutralizing these specific suppressive signals and blocking the pathogen’s attachment receptors can we hope to prevent the irreversible neural atrophy and life-threatening complications associated with neurosyphilis (**Table 5**) (131, 137).

## 10 Conclusion

Recent scholarship characterizes *Treponema pallidum* as a preeminent “stealth pathogen,” establishing that its neuroinvasive success is predicated upon a sophisticated biomechanical and molecular cascade. The literature consistently identifies the pathogen’s “naked” outer membrane—marked by a 100-fold lower density of integral proteins compared to enteric bacteria—and the stochastic antigenic variation of the TprK protein as core mechanisms for bypassing host innate and adaptive immune surveillance. Furthermore, there is a growing consensus that *Treponema pallidum* actively facilitates BBB traversal by triggering pro-inflammatory signals like IL-1β, which upregulates ADAMTS5 to degrade the endothelial glycocalyx. These findings are significant as they delineate how early systemic dissemination translates into persistent CNS involvement, providing a molecular roadmap for identifying novel drug targets, such as immunodominant extracellular loops, to disrupt immune evasion. However, current research is limited by a historical reliance on difficult rabbit models and the recentness of *in vitro* genetic engineering, which has only begun to overcome long-standing research bottlenecks regarding the spirochete’s metabolic fastidiousness. Additionally, many studies focus on serological cure via penicillin, often overlooking the irreversible neuro-inflammatory cascades that persist post-treatment. Future research should utilize sophisticated *in vitro* systems and neuroinformatics-based computational models to investigate the EndoMT and Rho GTPase signaling as drivers of vascular dysfunction. Addressing these specific suppressive signals is essential for developing therapeutic interventions that can arrest progressive neural atrophy and cognitive impairment before they become permanent.

## Glossary

ADAMTS5: a disintegrin and metalloproteinase with thrombospondin type 1 motif 5
AI: artificial intelligence
AIEgens: aggregation-induced emission luminogens
Akt: protein kinase B
AMR: antimicrobial resistance
BBB: blood-brain barrier
BFP: blue fluorescent protein
bp: base pair(s)
CNS: central nervous system
CSF: cerebrospinal fluid
DoxyPEP: doxycycline post-exposure prophylaxis
Doxy-PrEP: doxycycline-based pre-exposure prophylaxis
ECM: extracellular matrix
EndoMT: endothelial-to-mesenchymal transition
ER: endoplasmic reticulum
ERK: extracellular signal-regulated kinase
GBMSM: gay, bisexual, and other men who have sex with men
GFP: green fluorescent protein
GTPase: guanosine triphosphatase
hCMEC/D3: human cortical microvascular endothelial cells
HIF-1α: hypoxia-inducible factor 1-alpha
ICAM-1: intercellular adhesion molecule-1
IMP: intramembranous particle
IRE-1α: inositol-requiring enzyme 1 alpha
JAK1: Janus kinase 1
LamR: laminin receptor
LPS: lipopolysaccharide
MCP-1: monocyte chemoattractant protein-1
MSM: men who have sex with men
NF-κB: nuclear factor kappa B
PAMP: pathogen-associated molecular pattern
PEP: post-exposure prophylaxis
PERK: protein kinase R-like endoplasmic reticulum kinase
PI3K: phosphoinositide 3-kinase
PrEP: pre-exposure prophylaxis
STAT1: signal transducer and activator of transcription 1
STI: sexually transmitted infection
TGW: transgender women
TLR: Toll-like receptor
TPA: *Treponema pallidum* subsp. *pallidum*
TpCM-2: *Treponema pallidum* culture medium 2
TprK: *Treponema pallidum* repeat protein K
TROMP: rare outer membrane protein
VDRL: Venereal Disease Research Laboratory.

## References

[1] Fajemiroye JO Moreira ALE Ito CRM Costa EA Queiroz RM Ihayi OJ . Advancing syphilis research: Exploring new frontiers in immunology and pharmacological interventions. Venereology. (2023) 2:147–63. doi: 10.20944/preprints202308.2154.v1

[2] Schuenemann VJ Lankapalli AK Barquera R Nelson EA Hernández DI Acuña Alonzo V . Historic Treponema pallidum genomes from Colonial Mexico retrieved from archaeological remains. PloS NeglTrop Dis. (2018) 12:e0006447. doi: 10.1371/journal.pntd.0006447 29927932 PMC6013024

[3] Norris SJ . Polypeptides of Treponema pallidum: Progress toward understanding their structural, functional, and immunologic roles. Treponema Pallidum Polypeptide Research Group. Microbiol Rev. (1993) 57:750–79. doi: 10.1128/mr.57.3.750-779.1993 8246847 PMC372934

[4] Arora N Schuenemann VJ Jäger G Peltzer A Seitz A Herbig A . Origin of modern syphilis and emergence of a pandemic Treponema pallidum cluster. Nat Microbiol. (2016) 2:16245. doi: 10.1038/nmicrobiol.2016.245 27918528

[5] Fernandez MC Giacani L . Molecular and immunological strategies against Treponema pallidum infections. In: Cristaudo A Giuliani M , editors. Sexually Transmitted Infections. Springer International Publishing, Cham (2020). p. 391–411. doi: 10.1007/978-3-030-02200-6_21

[6] Bampi JVB Correa ME Bet GMDS Marchioro SB Simionatto S . Descriptive analysis of syphilis cases reported in Mato Grosso do Sul, Brazil identifies failure in treatment. Rev Soc Bras Med Trop. (2019) 52:e20180026. doi: 10.1590/0037-8682-0026-2018 31038618

[7] Beale MA Marks M Cole MJ Lee MK Pitt R Ruis C . Global phylogeny of Treponema pallidum lineages reveals recent expansion and spread of contemporary syphilis. Nat Microbiol. (2021) 6:1549–60. doi: 10.1038/s41564-021-01000-z 34819643 PMC8612932

[8] Tang Y Zhou Y He B Cao T Zhou X Ning L . Investigation of the immune escape mechanism of Treponema pallidum. Infection. (2023) 51:305–21. doi: 10.1007/s15010-022-01939-z 36260281

[9] Xu Q-Y Wang Y-J Lin L-R Liu L-L Yang T-C . The outer membrane lipoprotein Tp0136 stimulates human platelet activation and aggregation through PAR1 to enhance Gq/Gi signaling. Front Immunol. (2022) 13:818151. doi: 10.3389/fimmu.2022.818151 35296084 PMC8918515

[10] Radolf JD Deka RK Anand A Šmajs D Norgard MV Yang XF . Treponema pallidum, the syphilis spirochete: Making a living as a stealth pathogen. Nat Rev Microbiol. (2016) 14:744–59. doi: 10.1038/nrmicro.2016.141 27721440 PMC5106329

[11] Xiong S Liu Z Zhang X Huang S Ding X Zhou J . Resurgence of syphilis: Focusing on emerging clinical strategies and preclinical models. J Transl Med. (2023) 21:917. doi: 10.1186/s12967-023-04685-4 38105236 PMC10726518

[12] Predko I Bień N Hofmann J Skibińska M Narbutt J Sobolewska-Sztychny D . Coinfection of syphilis and HIV: Epidemiological evaluation at the Dermatology Department in Lodz, Poland. pdia. (2025) 42:354–60. doi: 10.5114/ada.2025.151144 41001142 PMC12458068

[13] Mahmud S Mohsin M Muyeed A Islam MM Hossain S Islam A . Prevalence of HIV and syphilis and their co-infection among men having sex with men in Asia: A systematic review and meta-analysis. Heliyon. (2023) 9:e13947. doi: 10.1016/j.heliyon.2023.e13947 36895383 PMC9988516

[14] Gállego-Lezáun C Arrizabalaga Asenjo M González-Moreno J Ferullo I Teslev A Fernández-Vaca V . Sífilis en hombres que tienen sexo con hombres: Una alarma para la detección de infección por VIH. Actas Dermo-Sifiliográficas. (2015) 106:740–5. doi: 10.1016/j.ad.2015.05.010 26188931

[15] Marra CM Maxwell CL Smith SL Lukehart SA Rompalo AM Eaton M . Cerebrospinal fluid abnormalities in patients with syphilis: Association with clinical and laboratory features. J Infect Dis. (2004) 189:369–76. doi: 10.1086/381227 14745693

[16] Marra CM Maxwell CL Tantalo L Eaton M Rompalo AM Raines C . Normalization of cerebrospinal fluid abnormalities after neurosyphilis therapy: Does HIV status matter? Clin Infect Dis. (2004) 38:1001–6. doi: 10.1086/382532 15034833

[17] LaFond RE Lukehart SA . Biological basis for syphilis. Clin Microbiol Rev. (2006) 19:29–49. doi: 10.1128/cmr.19.1.29-49.2006 16418521 PMC1360276

[18] Edmondson DG DeLay BD Kowis LE Norris SJ . Parameters affecting continuous *in vitro* culture of Treponema pallidum strains. mBio. (2021) 12:e03536-20. doi: 10.1128/mbio.03536-20 33622721 PMC8545124

[19] Barquera R Sitter TL Kirkpatrick CL Ramirez DA Kocher A Spyrou MA . Ancient genomes reveal a deep history of Treponema pallidum in the Americas. Nature. (2025) 640:186–93. doi: 10.1038/s41586-024-08515-5 39694065 PMC11964931

[20] Edmondson DG Hu B Norris SJ . Long-term *in vitro* culture of the syphilis spirochete Treponema pallidum subsp. pallidum. mBio. (2018) 9:e01153-18. doi: 10.1136/bmj.280.6210.303 29946052 PMC6020297

[21] Phan A Romeis E Tantalo L Giacani L . *In vitro* transformation and selection of Treponema pallidum subsp. pallidum. Curr Protocol. (2022) 2:e507. doi: 10.1002/cpz1.507 PMC9389596 35976045

[22] Cruz AR Ramirez LG Zuluaga AV Pillay A Abreu C Valencia CA . Immune evasion and recognition of the syphilis spirochete in blood and skin of secondary syphilis patients: Two immunologically distinct compartments. PloS NeglTrop Dis. (2012) 6:e1717. doi: 10.1371/journal.pntd.0001717 22816000 PMC3398964

[23] O’Shea JG . ‘Two minutes with Venus, two years with Mercury’-Mercury as an antisyphilitic chemotherapeutic agent. J R Soc Med. (1990) 83:392–5. doi: 10.1177/014107689008300619 PMC1292694 2199676

[24] Williams KJ . The introduction of ‘chemotherapy’ using arsphenamine - the first magic bullet. J R Soc Med. (2009) 102:343–8. doi: 10.1258/jrsm.2009.09k036 PMC2726818 19679737

[25] Bosch F Rosich L . The contributions of Paul Ehrlich to pharmacology: A tribute on the occasion of the centenary of his Nobel Prize. Pharmacology. (2008) 82:171–9. doi: 10.1159/000149583 18679046 PMC2790789

[26] Christensen SB . Drugs that changed society: History and current status of the early antibiotics: Salvarsan, sulfonamides, and β-lactams. Molecules. (2021) 26:6057. doi: 10.3390/molecules26196057 34641601 PMC8512414

[27] Tsay CJ . Julius Wagner-Jauregg and the legacy of malarial therapy for the treatment of general paresis of the insane. Yale J Biol Med. (2013) 86:245–54. PMC3670443 23766744

[28] O’leary PA . Penicillin in the treatment of syphilis. Med Clin North Am. (1946) 30:895–902. doi: 10.1016/S0025-7125(16)35928-4 20992948

[29] Karamanou M Kyriakis K Tsoucalas G Androutsos G . Hallmarks in history of syphilis therapeutics. Infez Med. (2013) 21:317–9. 24335464

[30] Chen J Huang J Liu Z Xie Y . Treponema pallidum outer membrane proteins: Current status and prospects. Pathog Dis. (2022) 80:ftac023. doi: 10.1093/femspd/ftac023 35869970

[31] Blanco DR Miller JN Lovett MA . Surface antigens of the syphilis spirochete and their potential as virulence determinants. Emerg Infect Dis. (1997) 3:11–20. doi: 10.3201/eid0301.970102 9126440 PMC2627599

[32] Radolf JD Kumar S . The Treponema pallidum outer membrane. In: Adler B , editor. Spirochete Biology: The Post Genomic Era, vol. 415 . Springer International Publishing, Cham (2017). p. 1–38. 10.1007/82_2017_44 PMC5924592 28849315

[33] Wu S Luo L Ye F Wang Y Li D . Comprehensive overview of Treponema pallidum outer membraneproteins. CPPS. (2024) 25:604–12. doi: 10.1016/j.jece.2026.121209 38661035

[34] Radolf JD Norgard MV Schulz WW . Outer membrane ultrastructure explains the limited antigenicity of virulent Treponema pallidum. Proc Natl Acad Sci USA. (1989) 86:2051–5. doi: 10.1007/82_2017_44 2648388 PMC286845

[35] Cox DL Chang P McDowall AW Radolf JD . The outer membrane, not a coat of host proteins, limits antigenicity of virulent Treponema pallidum. Infect Immun. (1992) 60:1076–83. doi: 10.1128/iai.60.3.1076-1083.1992 1541522 PMC257596

[36] Molini B Fernandez MC Godornes C Vorobieva A Lukehart SA Giacani L . B-cell epitope mapping of TprC and TprD variants of Treponema pallidum subspecies informs vaccine development for human treponematoses. Front Immunol. (2022) 13:862491. doi: 10.3389/fimmu.2022.862491 35422800 PMC9001972

[37] Li W Li S Wang J Yu M Yang H He Z . The outer membrane protein Tp92 of Treponema pallidum delays human neutrophil apoptosis via the ERK , PI3K / Akt , and NF‐κB pathways. Mol Microbiol. (2023) 120:684–701. doi: 10.1016/j.ijmm.2020.151416 37718557

[38] Haynes AM Fernandez M Romeis E Mitjà O Konda KA Vargas SK . Transcriptional and immunological analysis of the putative outer membrane protein and vaccine candidate TprL of Treponema pallidum. PLOS Neglected Tropical Diseases. (2021) 15:e0008812. doi: 10.1371/journal.pntd.0008812 PMC7864442 33497377

[39] Delgado KN Caimano MJ Orbe IC Vicente CF La Vake CJ Grassmann AA . Immunodominant extracellular loops of Treponema pallidum FadL outer membrane proteins elicit antibodies with opsonic and growth-inhibitory activities. PloS Pathog. (2024) 20:e1012443. doi: 10.1371/journal.ppat.1012443 39715273 PMC11761103

[40] Mukaremera L Lee KK Mora-Montes HM Gow NAR . Candida albicans yeast, pseudohyphal, and hyphal morphogenesis differentially affects immune recognition. Front Immunol. (2017) 8:629. doi: 10.3389/fimmu.2017.00629 28638380 PMC5461353

[41] Hernández-Chávez M Pérez-García L Niño-Vega G Mora-Montes HF . Fungal Strategies to Evade the Host Immune Recognition. Journal of Fungi. (2017) 3:51. doi: 10.3390/jof3040051 PMC5753153 29371567

[42] Liu A Giacani L Tuero I Klausner JD . Syphilis pathogenesis: Host immune response vs pathogen immune evasion. J Infect Dis. (2025) 232:760–6. doi: 10.1093/infdis/jiaf388 40708500 PMC12526937

[43] Romeis E Lieberman NAP Molini B Tantalo LC Chung B Phung Q . Treponema pallidum subsp. pallidum with an artificially impaired TprK antigenic variation system is attenuated in the rabbit model of syphilis. PloS Pathog. (2023) 19:e1011259. doi: 10.1371/journal.ppat.1011259 36940224 PMC10063172

[44] Lin MJ Haynes AM Addetia A Lieberman NAP Phung Q Xie H . Longitudinal TprK profiling of *in vivo* and *in vitro*-propagated Treponema pallidum subsp. pallidum reveals accumulation of antigenic variants in absence of immune pressure. PloS NeglTrop Dis. (2021) 15:e0009753. doi: 10.1371/journal.pntd.0009753 34492041 PMC8480903

[45] Sun P Liu S Zeng Q Li W Li S Wang Z . The regulatory mechanisms of Treponema pallidum enolase on macrophages: From enzymatic activity to signal transduction. FASEB J. (2025) 39:e70801. doi: 10.2217/epi.15.38 40600943 PMC12219466

[46] Wang YJ Xu QY Ye WM Yi DY Zheng XQ Xie L . Treponema pallidum Promotes the Polarization of M2 Subtype Macrophages to M1 Subtype Mediating the Apoptosis and Inhibiting the Angiogenesis of Human Umbilical Vein Endothelial Cells. ACS Infectious Diseases. (2023) 9:2548–2559. doi: 10.1021/acsinfecdis.3c00401 37983134

[47] Liu Z Zhang X Xiong S Huang S Ding X Xu M . Endothelial dysfunction of syphilis: Pathogenesis. Acad Dermatol Venereol. (2024) 38:1478–90. doi: 10.1111/jdv.19899 38376088

[48] Gao ZX Liu LL Lin LR Tong ML Liu F Yang TC . Treponema pallidum induces the secretion of HDVSMC inflammatory cytokines to promote the migration and adhesion of THP-1 cells. Front Cell Infect Microbiol. (2019) 9:220. doi: 10.3389/fcimb.2019.00220 31293985 PMC6598120

[49] Liu D Tong ML Lin Y Liu LL Lin LR Yang TC . Insights into the genetic variation profile of tprK in Treponema pallidum during the development of natural human syphilis infection. PloS NeglTrop Dis. (2019) 13:e0007621. doi: 10.1371/journal.pntd.0007621 31329597 PMC6675121

[50] Liu D Liu LL Zheng XQ Chen R Lin LR Yang TC . Genetic profiling of the full-length tprK gene in patients with primary and secondary syphilis. Microbiol Spectr. (2023) 11:e04931-22. doi: 10.1128/spectrum.04931-22 37036342 PMC10269439

[51] LaFond RE Molini BJ Van Voorhis WC Lukehart SA . Antigenic variation of TprK V regions abrogates specific antibody binding in syphilis. Infect Immun. (2006) 74:6244–51. doi: 10.1128/iai.00827-06 16923793 PMC1695500

[52] Reid TB Molini BJ Fernandez MC Lukehart SA . Antigenic variation of TprK facilitates development of secondary syphilis. Infect Immun. (2014) 82:4959–67. doi: 10.1128/iai.02236-14 25225245 PMC4249288

[53] Giacani L Molini BJ Kim EY Godornes BC Leader BT Tantalo LC . Antigenic variation in Treponema pallidum: TprK sequence diversity accumulates in response to immune pressure during experimental syphilis. J Immunol. (2010) 184:3822–9. doi: 10.4049/jimmunol.0902788 20190145 PMC3042355

[54] Addetia A Lin MJ Phung Q Xie H Huang ML Ciccarese G . Estimation of full-length TprK diversity in Treponema pallidum subsp. pallidum. mBio. (2020) 11:e02726-20. doi: 10.1128/mbio.02726-20 33109767 PMC7593977

[55] Young H Ogilvie M . Treponema pallidum sub-species pallidum (Syphilis). In: Young H Ogilvie M , editors. Genitourinary Infections. Springer Netherlands, Dordrecht (1994). p. 288–301. doi: 10.1007/978-94-017-5080-6_12

[56] Schäfer H . Syphilis. Der Hautarzt. (2004) 55:112–9. doi: 10.1007/s00105-003-0608-0 14749871

[57] Sethi G . Syphilis. In: Lewis FM , editor. Ridley’s the Vulva. Hoboken, NJ: Wiley (2022). p. 78–82. doi: 10.1002/9781119755173.ch11

[58] Lu S Zheng K Wang J Xu M Xie Y Yuan S . Characterization of Treponema pallidum dissemination in C57BL/6 mice. Front Immunol. (2021) 11:577129. doi: 10.3389/fimmu.2020.577129 33488577 PMC7819853

[59] Thomas DD Navab M Haake DA Fogelman AM Miller JN Lovett MA . Treponema pallidum invades intercellular junctions of endothelial cell monolayers. Proc Natl Acad Sci USA. (1988) 85:3608–12. doi: 10.1073/pnas.85.10.3608 3285346 PMC280263

[60] Houston S Hof R Honeyman L Hassler J Cameron CE . Activation and proteolytic activity of the Treponema pallidum metalloprotease, Pallilysin. PloS Pathog. (2012) 8:e1002822. doi: 10.1371/journal.ppat.1002822 22910436 PMC3406077

[61] Riley BS Oppenheimer-Marks N Hansen EJ Radolf JD Norgard MV . Virulent Treponema pallidum activates human vascular endothelial cells. J Infect Dis. (1992) 165:484–93. doi: 10.1093/infdis/165.3.484 1347056

[62] Waugh S Ranasinghe A Gomez A Houston S Lithgow KV Eshghi A . Syphilis and the host: multi-omic analysis of host cellular responses to Treponema pallidum provides novel insight into syphilis pathogenesis. Front Microbiol. (2023) 14:1254342. doi: 10.3389/fmicb.2023.1254342 37795301 PMC10546344

[63] Waugh S Ranasinghe A Reynolds LA Cameron CE . Treponema pallidum induces pro-inflammatory cytokine secretion in macrophages and macrophage-endothelial co-cultures. Front Cell Infect Microbiol. (2025) 15:1681813. doi: 10.3389/fcimb.2025.1681813 41181330 PMC12575358

[64] Church B Wall E Webb JR Cameron CE . Interaction of Treponema pallidum, the syphilis spirochete, with human platelets. PloS One. (2019) 14:e0210902. doi: 10.1371/journal.pone.0210902 30657796 PMC6338379

[65] Zhou Y Xie Y Xu M . Potential mechanisms of Treponema pallidum breaching the blood-brain barrier. Biomedicine Pharmacotherapy. (2024) 180:117478. doi: 10.1016/j.biopha.2024.117478 39321510

[66] Chen Z Du F Zhang R Wu Q Lu Z Zhang RL . ADAMTS5 promotes permeability of the blood-brain barrier during Treponema pallidum subspecies pallidum invading the central nervous system. ACS Infect Dis. (2024) 10:1222–31. doi: 10.1021/acsinfecdis.3c00664 38536197

[67] Kim KS . Mechanisms of microbial traversal of the blood–brain barrier. Nat Rev Microbiol. (2008) 6:625–34. doi: 10.1038/nrmicro1952 18604221 PMC5206914

[68] Yu H Zhao S Yang K Gao K Lin T Ling P . Treponema pallidum’s neural invasion: from blood-brain barrier breach to immune sabotage. Infection and Immunity. (2026) 94:e00621–25. doi: 10.1128/iai.00621-25 PMC12890026 41563128

[69] Fadel A Hussain H Hernandez RJ Clichy Silva AM Estillas AA Hamad M . Mechanisms of neurosyphilis-induced dementia: insights into pathophysiology. Neurol Int. (2024) 16:1653–65. doi: 10.3390/neurolint16060120 39728746 PMC11679807

[70] Luo X Zhang X Gan L Zhou C Zhao T Zeng T . The outer membrane protein Tp92 of Treponema pallidum induces human mononuclear cell death and IL‐8 secretion. J Cell Mol Medi. (2018) 22:6039–54. doi: 10.1016/j.micpath.2017.06.046 30596396 PMC6237608

[71] Zhou X Tang Y Cao T Ning L Li Y Xie X . Treponema pallidum lipoprotein Tp0768 promotes the migration and adhesion of THP‐1 cells to vascular endothelial cells through stress of the endoplasmic reticulum and the NF‐κB/HIF‐1α pathway. Molecular Microbiology. (2023) 119:86–100. doi: 10.1111/mmi.15010 36480422

[72] Cao T Li Y Zhou X Tang Y He B Cao Q . Treponema pallidum recombinant protein Tp0768 enhances the ability of HUVECs to promote neutrophil chemotaxis through the TLR2/ER stress signaling pathway. Journal of Leukocyte Biology. (2024) 116:1045–1053. doi: 10.1093/jleuko/qiae114 PMC11531811 38748684

[73] Primus S Rocha SC Giacani L Parveen N . Identification and functional assessment of the first placental adhesin of Treponema pallidum that may play critical role in congenital syphilis. Front Microbiol. (2020) 11:621654. doi: 10.3389/fmicb.2020.621654 33408711 PMC7779807

[74] He Y Chen D Fu Y Huo X Zhao F Yao L . Immunization with Tp0954, an adhesin of Treponema pallidum, provides protective efficacy in the rabbit model of experimental syphilis. Front Immunol. (2023) 14:1130593. doi: 10.3389/fimmu.2023.1130593 36993963 PMC10042077

[75] Singh AE Romanowski B . Syphilis: review with emphasis on clinical, epidemiologic, and some biologic features. Clin Microbiol Rev. (1999) 12:187–209. doi: 10.1128/cmr.12.2.187 10194456 PMC88914

[76] Pearce L Brown WH . A study of the relation of Treponema pallidum to lymphoid tissues in experimental syphilis. J Exp Med. (1922) 35:39–62. doi: 10.1084/jem.35.1.39 19868586 PMC2180240

[77] Kaminiów K Kiołbasa M Pastuszczak M . The Significance of the Cell-Mediated Host Immune Response in Syphilis. Microorganisms. (2024) 12:2580. doi: 10.3390/microorganisms12122580 PMC11677580 39770782

[78] Miron MI Diaconu CC Constantinescu TC Tatu AL . The multisystemic nature of syphilis: challenges in diagnosis. AnnalsARSciMed. (2025) 6:43–52. doi: 10.56082/annalsarscimed.2025.1.43

[79] Wu S Ye F Wang Y Li D . Neurosyphilis: insights into its pathogenesis, susceptibility, diagnosis, treatment, and prevention. Front Neurol. (2024) 14:1340321. doi: 10.3389/fneur.2023.1340321 38274871 PMC10808744

[80] Madhusudhan M . Neurosyphilis. Neurol India. (2009) 57:233. doi: 10.4103/0028-3886.53259 19587460

[81] Kaur B Khanna D . A narrative review of the many psychiatric manifestations of neurosyphilis: the great imitator. Cureus. (2023) 15:e44866. doi: 10.7759/cureus.44866 37814742 PMC10560128

[82] Cui L Xu Z Hou H . Diagnosis and treatment of spinal syphilitic gumma: a case report. Front Neurol. (2020) 10:1352. doi: 10.3389/fneur.2019.01352 32010040 PMC6974620

[83] Huang JL . 梅毒螺旋体黏附蛋白与宿主细胞间的作用研究进展. 皮肤性病诊疗学杂志. (2025) 32:215. doi: 10.3969/j.issn.1674-8468.2025.03.010

[84] Houston S Gomez A Geppert A Goodyear MC Cameron CE . In-depth proteome coverage of *in vitro*-cultured Treponema pallidum and quantitative comparison analyses with *in vivo*-grown treponemes. J Proteome Res. (2024) 23:1725–43. doi: 10.1021/acs.jproteome.3c00891 38636938 PMC11077495

[85] Bosák J Hrala M Andrla P Vrbová E Pospíšilová P Šmajs D . In vitro analysis of seven syphilis-causing Treponema pallidum strains revealed inherent growth rate differences. Scientific Reports. (2025) 15:33240. doi: 10.1038/s41598-025-18827-9 PMC12475411 41006811

[86] Edmondson DG De Lay BD Hanson BM Kowis LE Norris SJ . Clonal isolates of Treponema pallidum subsp. pallidum Nichols provide evidence for the occurrence of microevolution during experimental rabbit infection and *in vitro* culture. PloS One. (2023) 18:e0281187. doi: 10.1371/journal.pone.0281187 36917571 PMC10013896

[87] Grillová L Romeis E Lieberman NAP Tantalo LC Xu LH Molini B . Bright New Resources for Syphilis Research: Genetically Encoded Fluorescent Tags for Treponema pallidum and Sf1Ep Cells. Molecular Microbiology. (2024) 122:455–464. doi: 10.1111/mmi.15304 PMC11479824 39115038

[88] De Lay BD Cameron TA De Lay NR Norris SJ Edmondson DG . Comparison of transcriptional profiles of Treponema pallidum during experimental infection of rabbits and *in vitro* culture: highly similar, yet different. PloS Pathog. (2021) 17:e1009949. doi: 10.1371/journal.ppat.1009949 34570834 PMC8525777

[89] Zuo W Xiao Y Xiang Q Xiao S Xie Y . Participants in Treponema pallidum pathogenesis: progress in functional proteins. Front Immunol. (2025) 16:1632677. doi: 10.3389/fimmu.2025.1632677 40933996 PMC12417408

[90] Grillová L Carrami EM Roberts-Sengier W Thomson NR . High quality transcriptome profiling confirms the transcriptional landscape of Treponema pallidum subsp. pallidum. Sci Rep. (2025) 15:23272. doi: 10.1038/s41598-025-06583-9 PMC12223175 40604007

[91] Karimi MA Paryan M Behrouzian Fard G Sadeghian H Zarrinfar H Hosseini Bafghi M . Challenges and opportunities in the application of CRISPR-Cas9: a review on genomic editing and therapeutic potentials. Med Princ Pract. (2025) 35:1–17. doi: 10.1159/000547334 40675140 PMC12503735

[92] Romeis E Tantalo L Lieberman N Phung Q Greninger A Giacani L . Genetic engineering of Treponema pallidum subsp. pallidum, the syphilis spirochete. PLoS Pathog. (2021) 17:e1009612. doi: 10.1371/journal.ppat.1009612 PMC8284648 34228757

[93] Patrick R . Advancing CRISPR/Cas technology: overcoming challenges to create the ultimate genome editing tool. (2024). doi: 10.5281/ZENODO.14559526

[94] Toddes CA . An accessible intersectional transgenic single-vector CRISPR/Cas9 platform for precise gene editing and functional analysis. eNeuro. (2024) 11:ENEURO.0264-24.2024. doi: 10.1523/eneuro.0264-24.2024 39103226 PMC11302499

[95] Ehrke-Schulz E Schiwon M Hagedorn C Ehrhardt A . Establishment of the CRISPR/Cas9 system for targeted gene disruption and gene tagging. In: Kaufmann M Klinger C Savelsbergh A , editors. Functional Genomics, vol. 1654 . Springer New York, New York, NY (2017). p. 165–76. 10.1007/978-1-4939-7231-9_11 28986789

[96] Delgado KN Vicente CF Hennelly CM Aghakhanian F Parr JB Claffey KP . Development and utilization of Treponema pallidum expressing green fluorescent protein to study spirochete-host interactions and antibody-mediated clearance: expanding the toolbox for syphilis research. mBio. (2025) 16:e03253-24. doi: 10.1128/mbio.03253-24 PMC11708019 39611839

[97] Huang J Zheng J Chen X Lin W Zhao M Xie Q . In Situ High‐Resolution Imaging of Live Treponema pallidum Using Catalyst‐Free Bioorthogonal Strategy. Aggregate. (2025) 6:e70154. doi: 10.1002/agt2.70154

[98] Pereira LE Katz SS Sun Y Mills P Taylor W Atkins P . Successful isolation of Treponema pallidum strains from patients’ cryopreserved ulcer exudate using the rabbit model. PloS One. (2020) 15:e0227769. doi: 10.1371/journal.pone.0227769 31929602 PMC6957173

[99] Edmondson DG Norris SJ . *In vitro* cultivation of the syphilis spirochete Treponema pallidum. Curr Protocol. (2021) 1:e44. doi: 10.1002/cpz1.44 33599121 PMC7986111

[100] Houston S Gomez A Geppert A Eshghi A Smith DS Waugh S . Deep proteome coverage advances knowledge of Treponema pallidum protein expression profiles during infection. Sci Rep. (2023) 13:18259. doi: 10.1038/s41598-023-45219-8 37880309 PMC10600179

[101] Fraser CM Norris SJ Weinstock GM White O Sutton GG Dodson R . Complete genome sequence of Treponema pallidum, the syphilis spirochete. Science. (1998) 281:375–88. doi: 10.1073/pnas.061029298 9665876

[102] Pham MD Ong JJ Anderson DA Drummer HE Stoové M . Point-of-care diagnostics for diagnosis of active syphilis infection: Needs, challenges and the way forward. IJERPH. (2022) 19:8172. doi: 10.3390/ijerph19138172 35805831 PMC9265885

[103] Jancar N Simões M Gonçalves F Duro J Aguiar P . Neurosyphilis: the great imitator. Cureus. (2022) 14:e32747. doi: 10.7759/cureus.32747 36686118 PMC9851570

[104] Chow F . Neurosyphilis. Continuum. (2021) 27:1018–39. doi: 10.1212/con.0000000000000982 34623102

[105] Batayneh O Deblois K Abu-Jaradeh O Zainah H . A case of neurosyphilis and review of manifestations, diagnosis, and treatment. R I Med J (2013). (2022) 105:9–11. 35081181

[106] Zezetko A Stallings M Pastis I . Deciphering the great imitator: syphilis and neurosyphilis. Cureus. (2024) 16:e54563. doi: 10.7759/cureus.54563 38516442 PMC10955426

[107] Treger RS Menza TW Truong TT Lieberman JA . Advances in syphilis diagnostics to address the 21st-century epidemic. Clin Chem. (2025) 71:935–48. doi: 10.1093/clinchem/hvaf072 40510004 PMC12405703

[108] Jaiswal AK Rodrigues Gomes LG Ferreira Maciel De Oliveira A De Castro Soares S Azevedo V . The critical role of penicillin in syphilis treatment and emerging resistance challenges. Diseases. (2025) 13:41. doi: 10.3390/diseases13020041 39997048 PMC11854480

[109] Hamill MM Ghanem KG Tuddenham S . State-of-the-art review: neurosyphilis. Clin Infect Dis. (2024) 78:e57–68. doi: 10.1093/cid/ciad437 37593890

[110] Galindo J Mier JF Miranda CA Rivas JC . Neurosífilis: Un problema antiguo que no pierde actualidad. Rev Colombiana Psiquiatría. (2017) 46:69–76. doi: 10.1016/j.rcp.2017.05.002 29037341

[111] Taylor MM Nurse-Findlay S Zhang X Hedman L Kamb ML Broutet N . Estimating benzathine penicillin need for the treatment of pregnant women diagnosed with syphilis during antenatal care in high-morbidity countries. PloS One. (2016) 11:e0159483. doi: 10.1371/journal.pone.0159483 27434236 PMC4951037

[112] Xiao H Liu D Li Z Zheng R Li Z Hou J . Comparison of doxycycline and benzathine penicillin G for the treatment of early syphilis. Acta Dermatovenerologica Croatica. (2017) 25:107–111. 28871924

[113] Shenoy ES Macy E Rowe T Blumenthal KG . Evaluation and management of penicillin allergy: A review. JAMA. (2019) 321:188. doi: 10.1001/jama.2018.19283 30644987

[114] Fica A Muñoz D Rojas T Sanzana C Muñoz C . Penicillin desensitization in allergic pregnant women with syphilis. Report of two cases. Rev méd Chile. (2020) 148:344–8. doi: 10.4067/s0034-98872020000300344 32730379

[115] Silverberg B Moyers A Hinkle T Kessler R Russell NG . 2021 CDC update: Treatment and complications of sexually transmitted infections (STIs). Venereology. (2022) 1:23–46. doi: 10.3390/venereology101000430654563

[116] Newman L Kamb M Hawkes S Gomez G Say L Seuc A . Global estimates of syphilis in pregnancy and associated adverse outcomes: Analysis of multinational antenatal surveillance data. PloS Med. (2013) 10:e1001396. doi: 10.1371/journal.pmed.1001396 23468598 PMC3582608

[117] Ferreira AM Pedrosa DV . Gestational syphilis: The importance of proper treatment in the prevention of congenital syphilis. In: Seven Editora. São José dos Pinhais, PR, Brazil: Seven Editora (2023). doi: 10.56238/colleinternhealthscienv1-127

[118] De Santis M De Luca C Mappa I Spagnuolo T Licameli A Straface G . Syphilis infection during pregnancy: Fetal risks and clinical management. Infect Dis Obstetrics Gynecology. (2012) 2012:1–5. doi: 10.1155/2012/430585 22829747 PMC3398589

[119] Dallé J Ramos MC Jimenez MF Escobar FG Antonello VS . Oral Desensitization to Penicillin for the Treatment of Pregnant Women with Syphilis: A Successful Program. Revista Brasileira de Ginecologia e Obstetrícia. (2018) 40:43–46. doi: 10.1055/s-0037-1606274 PMC10416178 28859210

[120] Coyle M Depcinski S Thirumoorthi M . Prevention of congenital syphilis using ceftriaxone in a woman with Stevens–Johnson syndrome reaction to penicillin: A case report. Case Rep Women’s Health. (2022) 36:e00446. doi: 10.1016/j.crwh.2022.e00446 36072694 PMC9441298

[121] Sølling J From E Mogensen CE . The role of immune complexes in early syphilis and in the Jarisch-Herxheimer reaction. Acta Dermato-Venereologica. (1982) 62:325–329. doi: 10.2340/0001555562325329 6183866

[122] Cornelisse VJ Riley B Medland NA . Australian consensus statement on doxycycline post‐exposure prophylaxis (doxy‐PEP) for the prevention of syphilis, chlamydia and gonorrhoea among gay, bisexual and other men who have sex with men. Med J Aust. (2024) 220:381–6. doi: 10.5694/mja2.52258 38479437

[123] Bachmann LH Barbee LA Chan P Reno H Workowski KA Hoover K . CDC clinical guidelines on the use of doxycycline postexposure prophylaxis for bacterial sexually transmitted infection prevention, United States, 2024. MMWR Recomm Rep. (2024) 73:1–8. doi: 10.15585/mmwr.rr7302a1 38833414 PMC11166373

[124] Doan L Joslyn P Sung L Breiner T Marotta S Rastogi S . (2025). “ Assessing the efficacy of prolonged doxycycline PEP use in high-risk populations for STI prevention”, in: 29th Annual Research Day (Glassboro, NJ: Rowan University Libraries). doi: 10.31986/issn.2689-0690_rdw.stratford_research_day.186_2025

[125] Tran NK Goldstein ND Welles SL . Countering the rise of syphilis: A role for doxycycline post-exposure prophylaxis? Int J STD AIDS. (2022) 33:18–30. doi: 10.1177/09564624211042444 34565255 PMC8688295

[126] Wu E-L Pagkas-Bather J Hazra A Pho MT . DoxyPEP: opportunities for expansion. J Prim Care Community Health. (2025) 16:21501319251370497. doi: 10.1177/21501319251370497 40913480 PMC12414116

[127] Werner RN Schmidt AJ Potthoff A Spornraft-Ragaller P Brockmeyer NH German STI Society (DSTIG) . Stellungnahme der Deutschen STI‐Gesellschaft zur antibiotischen STI‐Prophylaxe mit Doxycyclin (Doxy‐PEP, Doxy‐PrEP): Position Statement of the German STI Society on the Prophylactic Use of Doxycycline to Prevent STIs (Doxy‐PEP, Doxy‐PrEP). Journal der Deutschen Dermatologischen Gesellschaft. (2024) 22:466–480. doi: 10.1111/ddg.15282_g 38123738

[128] Hall C . Increasing the prescribing of pre-exposure prophylaxis (PrEP) with a pre-exposure prophylaxis screening tool. In: University of St. Augustine for Health Sciences. San Marcos, CA: University of St. Augustine for Health Sciences (2022). doi: 10.46409/sr.ZIXA9889

[129] Golden MR Handsfield HH . Preexposure prophylaxis to prevent bacterial sexually transmitted infections in men who have sex with men. Sexually Transmitted Dis. (2015) 42:104–6. doi: 10.1097/olq.0000000000000235 25585070

[130] Cortés-Penfield NW Musher DM . Give penicillin or ceftriaxone: Neurosyphilis does not deal in absolutes. Clin Infect Dis. (2024) 78:1386–7. doi: 10.1093/cid/ciad629 37847217

[131] Waugh S Goodyear MC Gomez A Ranasinghe A Lithgow KV Falsafi R . Time-course transcriptomics reveals the impact of Treponema pallidum on microvascular endothelial cell function and phenotype. Front Microbiol. (2025) 16:1649738. doi: 10.3389/fmicb.2025.1649738 41122461 PMC12535989

[132] Luo X Zhang L Xie X Yuan L Shi Y Jiang Y . Phosphorylated vimentin-triggered fibronectin matrix disaggregation enhances the dissemination of Treponema pallidum subsp. pallidum across the microvascular endothelial barrier. PloS Pathog. (2024) 20:e1012483. doi: 10.1371/journal.ppat.1012483 39226326 PMC11398692

[133] Hu Y-T Wu K-X Wang X-T Zhao Y-Y Jiang X-Y Liu D . Treponema pallidum promoted microglia apoptosis and prevented itself from clearing by human microglia via blocking autophagic flux. PloS Pathog. (2023) 19:e1011594. doi: 10.1371/journal.ppat.1011594 37611054 PMC10446187

[134] Hayes KA Dressler JM Norris SJ Edmondson DG Jutras BL . A large screen identifies beta-lactam antibiotics which can be repurposed to target the syphilis agent. NPJ Antimicrob Resist. (2023) 1:4. doi: 10.1038/s44259-023-00006-3 38686211 PMC11057208

[135] Osias E Hung P Giacani L Stafylis C Konda KA Vargas SK . Investigation of syphilis immunology and Treponema pallidum subsp. pallidum biology to improve clinical management and design a broadly protective vaccine: study protocol. BMC Infectious Diseases. (2020) 20:444. doi: 10.1186/s12879-020-05141-0 PMC7309211 32576149

[136] Hawley KL Montezuma-Rusca JM Delgado KN Singh N Uversky VN Caimano MJ . Structural modeling of the Treponema pallidum outer membrane protein repertoire: A road map for deconvolution of syphilis pathogenesis and development of a syphilis vaccine. J Bacteriol. (2021) 203:e00082–21. doi: 10.1128/jb.00082-21 33972353 PMC8407342

[137] Lithgow KV Church B Gomez A Tsao E Houston S Swayne LA . Identification of the neuroinvasive pathogen host target, LamR, as an endothelial receptor for the Treponema pallidum adhesin Tp0751. mSphere. (2020) 5:e00195-20. doi: 10.1128/msphere.00195-20 32238570 PMC7113585

[138] Weng W Song B Zhang M Zhu W . Untargeted metabolomics in a prospective cross-sectional observational study reveals differences in plasma and cerebrospinal fluid between asymptomatic neurosyphilis and serofast syphilis. Medicine. (2025) 104:e45361. doi: 10.1097/md.0000000000045361 41189184 PMC12537089

[139] Jaiswal AK Jamal SB Gabriel Rodrigues Gomes L Profeta R Sales-Campos H Oliveira CJF . Neuroinformatics insights towards multiple neurosyphilis complications. Venereology. (2022) 1:135–60. doi: 10.3390/venereology101001030654563

